# A scoping review of outcome selection and accuracy of conclusions in complex digital health interventions for young people (2017–2023): methodological proposals for population health intervention research

**DOI:** 10.1186/s12916-025-04245-1

**Published:** 2025-07-02

**Authors:** Claire Collin, Clara Eyraud, Philippe Martin, Morgane Michel, Enora Le Roux, Corinne Alberti

**Affiliations:** 1 Université Paris Cité, Inserm, ECEVE, Paris, F-75010 France; 2 AP-HP, Nord-Université Paris Cité, Inserm, Hôpital Universitaire Robert Debré, Clinical Epidemiology Unit, CIC 1426, Paris, F-75019 France; 3 Institut National d’Etudes Démographiques (INED), UR14 - Sexual and Reproductive Health and Rights, Aubervilliers, F-93300 France; 4 University Center for Adolescent and Young Adult Health, Fondation Santé des Etudiants de France, Paris, F-75014 France

**Keywords:** Methodological research, Outcomes, Interventions, Population health intervention research, Scoping review

## Abstract

**Background:**

Determining the success of population health interventions often involves assessing multiple, multidimensional outcomes rather than a single one, which presents significant methodological challenges under the evidence-based medicine paradigm. This scoping review examines outcome selection, analysis, and interpretation, and the accuracy of conclusions in complex digital health interventions promoting health among adolescents and young adults (DHI-AYA).

**Methods:**

A comprehensive search of PubMed, EMBASE, ClinicalTrials.gov, PsycINFO, and CINAHL identified DHI-AYA implemented between 2017 and 2023. Studies were categorised by methodological choice regarding outcome hierarchical position: unique primary, multiple primary, or non-hierarchised outcomes. Outcomes were further classified into effectiveness, process, or economic categories. The authors’ conclusions on intervention success were compared with conclusions drawn by the research team based on the reported outcome analysis strategy. Secondly, four analytical strategies were applied to a subset of selected interventions to illustrate the impact of outcome hierarchical position and number on conclusions about intervention success.

**Results:**

Analysis of 100 studies linked to 26 DHI-AYA identified 251 distinct outcomes: 164 effectiveness, 78 process, and 9 economic outcomes. Seven interventions were evaluated using a unique primary outcome, 10 using multiple primary outcomes, and 9 using multiple non-hierarchised outcomes. Primary and secondary outcomes were predominantly effectiveness endpoints. The research team reclassified nine interventions (35%) deemed successful by authors as non-conclusive due to statistically conflicting results across outcomes. Most interventions deemed non-conclusive by the research team were evaluated using non-hierarchised outcomes (7/10, 70%). The choice of outcome analysis strategy substantially affected conclusions on intervention success.

**Conclusions:**

Discrepancies in intervention success assessments highlight the need for enhanced transparency, robustness, and trustworthiness in conclusion-drawing processes. In response, five methodological proposals are formulated: (1) developing core outcome sets specific to population health intervention research (PHIR), (2) collaboratively selecting multidimensional outcomes through a steering committee that accounts for stakeholder preferences and existing theoretical models, (3) exploring multi-criteria decision analysis and consensus-driven methods to transparently combine outcomes, (4) enhancing methodological reporting through intervention development and evaluation to improve scientific integrity and reproducibility, and (5) increasing PHIR expert involvement in ethics, funding, and evaluation committees to improve recognition of evidence produced in this field.

**PROSPERO Registration number:**

CRD42023401979.

**Supplementary Information:**

The online version contains supplementary material available at 10.1186/s12916-025-04245-1.

## Background

Health research spans a continuum of fields from (pre)clinical sciences to population health intervention research (PHIR), each characterised by distinct focuses and methodologies [1]. While clinical research has long adhered to standardised practices, notably randomised controlled trials often centred on a unique primary outcome under the evidence-based medicine (EBM) paradigm [2, 3], PHIR is still developing and refining its methodological framework [4].

The UK Medical Research Council (MRC) has significantly advanced PHIR methodology [5] through guidance for developing and evaluating complex interventions [6], which constitute the majority of PHIR studies [7]. This guidance outlines four research phases—development, feasibility, evaluation (encompassing efficacy or effectiveness, process and efficiency evaluations), and implementation—alongside six core elements to consider throughout these phases (i.e. context, programme theory, stakeholder engagement, key uncertainties, intervention refinement, economic considerations). While providing a valuable framework for evaluating PHIR, the guidance offers limited direction regarding the practical application of evaluation methodologies [6].

One key methodological challenge in PHIR is the selection, analysis, and interpretation of multiple outcomes. The use of multiple outcomes is recommended to assess the full range of intervention effects across several dimensions, including effectiveness, process, efficiency, and implementation contexts [6]. While this approach aims to support conclusions about interventions’ success and deployment [8], it raises several methodological issues: increased risk of type I errors [9], complex sample size calculations [10], and potential interpretation difficulties when measured outcomes yield conflicting results [11].

These methodological issues are particularly reported in the evaluation of digital health interventions, which are emerging as a growing focus in PHIR [12]. Digital health interventions are typically considered complex interventions, composed of multiple interactive components, often targeting several health behaviours simultaneously and implemented across diverse contextual settings [13]. They offer broad reach at a relatively low cost [14] and are particularly well-suited to promote behaviour change among adolescents and young adults, a group that extensively uses digital platforms for socialising and accessing health information [15]. The limited availability of robust evidence hinders the widespread implementation of evidence-based digital health interventions, highlighting the need for further methodological research [16, 17].

This study aims to examine the methodological decisions made by researchers in selecting, analysing, and interpreting outcomes to determine the success of complex digital interventions promoting health among adolescents and young adults. While these methodological issues extend beyond digital interventions, they serve as an illustrative example for broader applications within PHIR.

## Methods

A scoping review was conducted following PRISMA guidelines [18] (see Additional file 1: Tables S1–S2 [18] for the completed PRISMA 2020 Checklists) and registered with PROSPERO (CRD42023401979). The review examined digital health interventions (DHIs) promoting health among adolescents and young adults (AYA), focusing on the methods and outcomes used for evaluation and the impact of different methodological approaches on intervention conclusions.

### Eligibility of digital health interventions

Eligibility was assessed at the intervention level, identifying complex primary prevention or health promotion interventions for AYA aged 10 to 24, delivered exclusively via digital technologies (e.g. computer programmes, mobile applications, websites, social media, text messaging services, wearable devices) [19]. Interventions were classified as complex when comprising at least two distinct components (e.g. videos, quizzes, information sheets) and engaging users through multiple modalities, ranging from passive information delivery to interactive engagement with peers or healthcare professionals. To ensure the contemporary relevance of included technologies in a rapidly evolving digital landscape, interventions had to be implemented between 2017 and 2023, with 2017 specifically selected as the search start date following the publication of several seminal papers that established fundamental frameworks for digital health intervention design and evaluation [12, 13, 20–24]. Eligible interventions were required to report outcome results from an efficacy or effectiveness evaluation (evaluation phase) and from at least one additional MRC-defined research phase among development, feasibility, or implementation [6]. This approach aimed to identify interventions already adhering to the best methodological recommendations to explore gaps in their practical application rather than highlight the need for improved guideline adherence. Publications not in English or French were excluded.

### Search strategy

PubMed, EMBASE, ClinicalTrials.gov, PsycINFO, and CINAHL were searched on February 9th, 2023, with a last update on January 2nd, 2024. Five groups of relevant keywords were combined: “eHealth”, “intervention research”, “evaluation”, “health promotion and prevention”, and “adolescents and young adults”. The search strategy was iteratively developed with input from the research team and support from the university library (see Additional file 2 for detailed search strategies). For each eligible intervention, all related protocols, published articles, and preprints were retrieved through citation and reference searching, regardless of publication date. Ancillary studies were excluded [25].

### Selection process, data extraction, and risk-of-bias assessment

Two researchers (CC, CE) independently screened titles and abstracts, reviewed full texts, and extracted data using Covidence systematic review software (Veritas Health Innovation, Melbourne, Australia). Disagreements were resolved through discussion with a third researcher (ELR or PM).

Data were extracted using a standardised form (see Additional file 3), collecting the following: (1) intervention characteristics, including health topics, population demographics, types and number of digital technologies employed, and intervention components; (2) reported outcomes, including variable domain, specific measurement, metric and time points, method of aggregation [26], hierarchical position (primary, secondary, non-hierarchised), number of outcomes per position, and statistical significance of the result for each outcome (statistically significant or not statistically significant at level 5% with bilateral testing potentially corrected for multiple testing, or not tested); (3) authors’ conclusions regarding their interventions, categorised into three groups: (i) success (S) in achieving intervention objectives, (ii) failure (F) in achieving objectives, or (iii) non-conclusive (NC) due to statistically conflicting results; and (4) risk of bias for individual studies, assessed using Cochrane’s tools for randomised controlled trials [27] and non-randomised studies [28].

Outcome hierarchical positions were extracted from study protocols or, when unavailable, from the *Methods* section of primary effectiveness papers.

### Data synthesis

#### Outcomes categorisation

Outcomes were categorised according to their nature and role in conclusions.

##### Nature of outcomes

Outcomes were classified into three categories: effectiveness (encompassing efficacy and effectiveness outcomes), process, and economic outcomes.

Effectiveness outcomes were grouped into clinical outcomes (e.g. pregnancy, body mass index), healthcare utilisation (e.g. HIV or sexually transmitted infections (STIs) testing), behavioural outcomes (e.g. condom use, substance use), psychosocial outcomes (e.g. stress, coping), and behavioural determinants (i.e. knowledge, attitudes, perceived barriers, outcomes expectancy, norms, self-efficacy, skills, and intentions) as identified in behavioural change theories [29].

Process outcomes were grouped according to the MRC guidance for process evaluation (i.e. dose, fidelity, reach, contextual factors), supplemented by the Proctor’s outcomes for implementation research (i.e. acceptability, adoption, appropriateness, feasibility, retention, perceived effectiveness, usability) [30, 31]. Additionally, safety, defined as participants feeling safe using the intervention, was included as a process outcome, consistent with its classification in the original publication [32].

Economic outcomes, as defined in this study, encompass both cost estimates (i.e. absolute costs) and cost-effectiveness metrics (i.e. incremental cost-effectiveness ratios (ICER), incremental-cost utility ratios (ICUR), and costs per disability-adjusted life year (DALY)).

##### Role in conclusions

Outcomes were classified as ‘used to conclude’ if mentioned in the justification of intervention success or failure, or ‘not used’ if only reported in *Results*.

#### Interventions categorisation

At the intervention level, studies were classified into three groups based on the outcomes defined in the effectiveness evaluation phase, distinguishing those using (A) a unique primary outcome (with or without secondary or additional non-hierarchised outcomes), (B) multiple primary outcomes (with or without secondary or additional non-hierarchised outcomes), or (C) multiple non-hierarchised outcomes.

### Data analysis

The analysis comprised the following: (1) a comparison between the authors’ conclusions about intervention success, as stated on the articles’ discussion or conclusion sections, and conclusions drawn by the research team based on the authors’ reported outcome analysis strategy for each intervention, and (2) a sub-analysis examining how different outcome analysis strategies (i.e. outcome hierarchical positions and numbers) may influence overall intervention conclusions.

First, the research team assigned their conclusions to each intervention, based on outcomes’ hierarchical position specified in the study protocols or *Methods* sections and results (effect size and statistical significance) reported in the *Results* sections. Interventions deemed successful by authors but unsuccessful by researchers were classified as spin. A spin is defined as “the intentional or unintentional specific reporting that unfaithfully reflects the nature and range of findings” [33]. Agreement percentages between authors’ and researchers’ conclusions were calculated.

Second, three sexual and reproductive health interventions were selected from the analysed articles to explore the influence of outcome analysis strategy on conclusions. The sub-analysis was restricted to effectiveness outcomes. The three interventions were purposefully selected to represent each of the three approaches to outcome analysis (i.e. use of a unique primary outcome (category A), multiple primary outcomes (category B), or multiple non-hierarchised outcomes (category C)), while being as comparable as possible on other characteristics, including type of digital technology, target population age, theoretical framework, and at least one shared effectiveness outcome (i.e. number of protected sex acts).

Four strategies reflecting varying outcome hierarchical positions and numbers, identified in the review and the existing literature [34], were applied to the three interventions:

1) Unique primary outcome, with conclusions based on this outcome.

2) Multiple non-hierarchised outcomes, with conclusions based on all measured outcomes, without prioritisation; this strategy required all outcomes to show significant improvement to deem the intervention successful.

For the following strategies 3 and 4, only a limited number of outcomes, comparable across the chosen interventions or considered important by the original authors, were included in determining success. Each selected outcome was assigned a score and a weight. The score was binary: 1 for improvement (i.e. statistically significant) or 0 for no improvement (i.e. not statistically significant). The weight was chosen between 0 and 1 (totalling 1). Each outcome’s weight was then multiplied by its score, and the total intervention score was calculated by summing all obtained products. Interventions scoring above 0.5 were classified as successful.

3) Multiple non-hierarchised outcomes equally weighted, with conclusions based on the equally weighted total score of the selected outcomes;

4) Multiple non-hierarchised outcomes differentially weighted, with conclusions based on the differentially weighted total score of the selected outcomes. One researcher (CC) assigned the weights according to the relevance of the outcomes to the health condition (Additional file 4: Table S3 [35]).

Results are presented using descriptive statistics. Categorical variables are expressed as counts and percentages, and numerical variables as medians with 1st and 3rd quartiles (med, (Q1-Q3)).

## Results

### Study selection

**Fig. 1:**
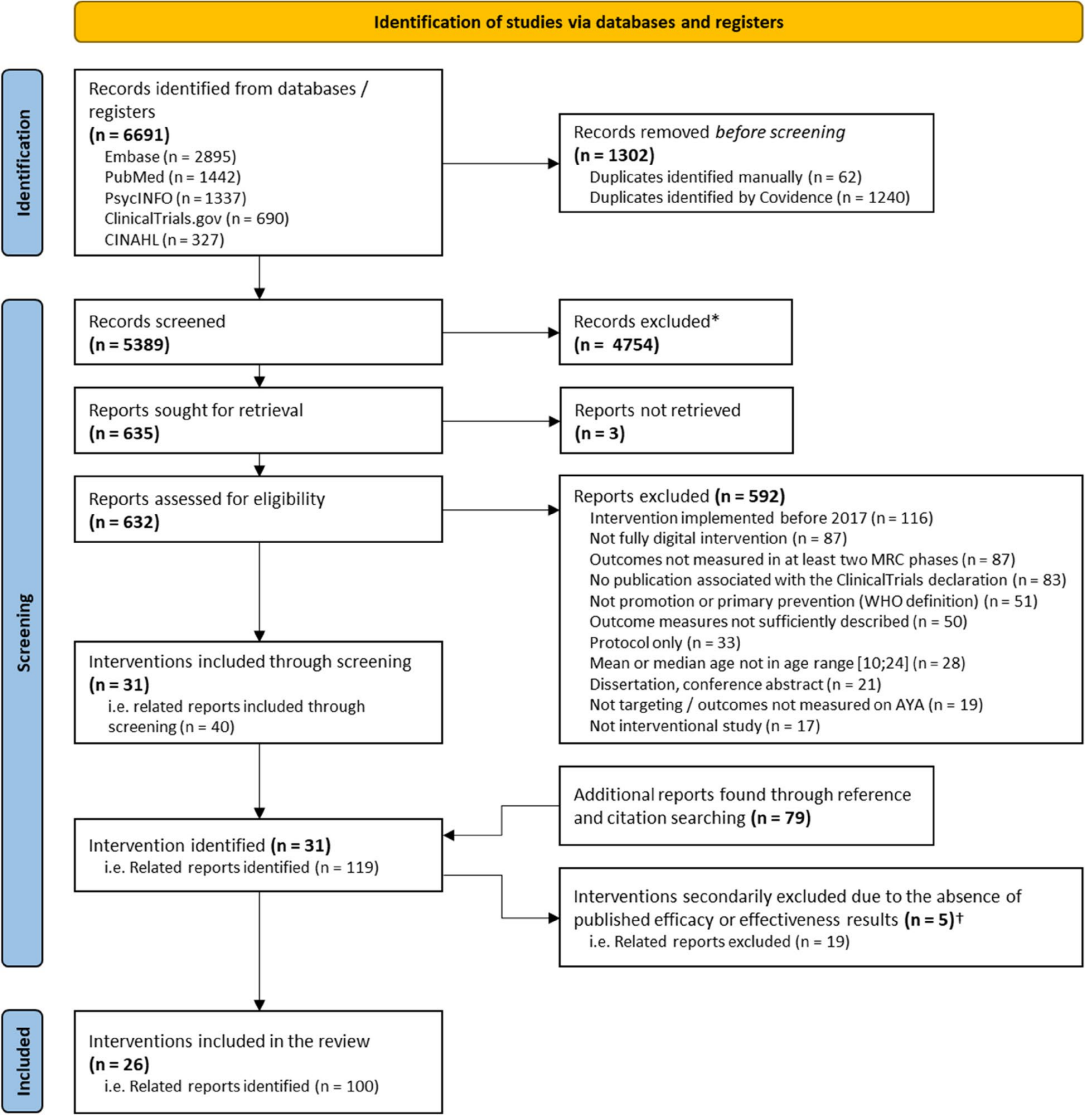
PRISMA flow diagram of literature search *Exclusion reasons are similar to those for full-text assessment; †These interventions were excluded because only efficacy or effectiveness evaluation protocols were available, which would have resulted in insufficient data for comprehensive analysis

From the 6691 records initially identified, 26 interventions described across 100 individual reports were analysed (Fig. 1, Additional file 5 for the full list of included reports [32, 36–134]). A median of four articles per intervention was included (Q1–Q3: 3–5).

### Intervention characteristics

Included interventions predominantly targeted sexual and reproductive health (13/26, 50%), were delivered through a dedicated mobile app or website (20/26, 77%), using a single technology (17/26, 65%), over a median duration of 3 months (Q1–Q3: 1–7). Nearly three-quarters of interventions were implemented and evaluated in daily-life settings (19/26, 73%) (Table 1). Most interventions were evaluated using individual or cluster randomised controlled trials (23/26, 88%), with the remainder using quasi-experimental pre-post designs (3/26, 12%).

**Table 1:** Distribution of characteristics for included interventions (*n* = 26)

General characteristics	All interventions
	*n* (%)*
Health topics	
Sexual and reproductive health	13 (50)
Substance use (alcohol, tobacco, drugs)	6 (23)
Mental health	5 (19)
Physical activity and/or nutrition	2 (8)
Main type of digital technology used in interventions	
Mobile app or website	20 (77)
Social media	3 (12)
Text-messaging services (e.g. SMS^x^, emails)	3 (12)
Number of digital technologies used in interventions	
Single technology	17 (65)
Multiple technologies	9 (35)
Recruitment setting	
Educational setting (school, university)	10 (38)
Online / Internet	6 (23)
Multi-setting†	6 (23)
Healthcare facilities	2 (8)
Other (community-based organisations, phone)	2 (8)
Implementation setting	
Daily life (online, home, etc.)	19 (73)
Educational setting (school, university)	7 (27)
Intervention categorisation	
Intervention evaluated using a unique primary outcome	7 (27)
Intervention evaluated using multiple primary outcomes	10 (38)
Intervention evaluated using non-hierarchised outcomes	9 (35)
Outcomes	med (Q1–Q3)
Number of reported outcomes per intervention‡	8 (5–11)
Effectiveness outcomes (*n* = 26)	5 (4–7)
Process outcomes (*n* = 17)	3 (2–6)
Economic outcomes (*n* = 3)	3 (3–4)

*Percentages may not add up to 100 due to rounding; ^*x*^*SMS:* Short Message Service; †: multi-setting refers to recruitment that occurred in at least two different settings, including educational settings, online or internet-based platforms, healthcare facilities, or other settings (e.g. community-based organisations, phone); ‡: number of outcomes regardless of outcome hierarchical position; excludes outcomes mentioned in protocols only

**Fig. 2:**
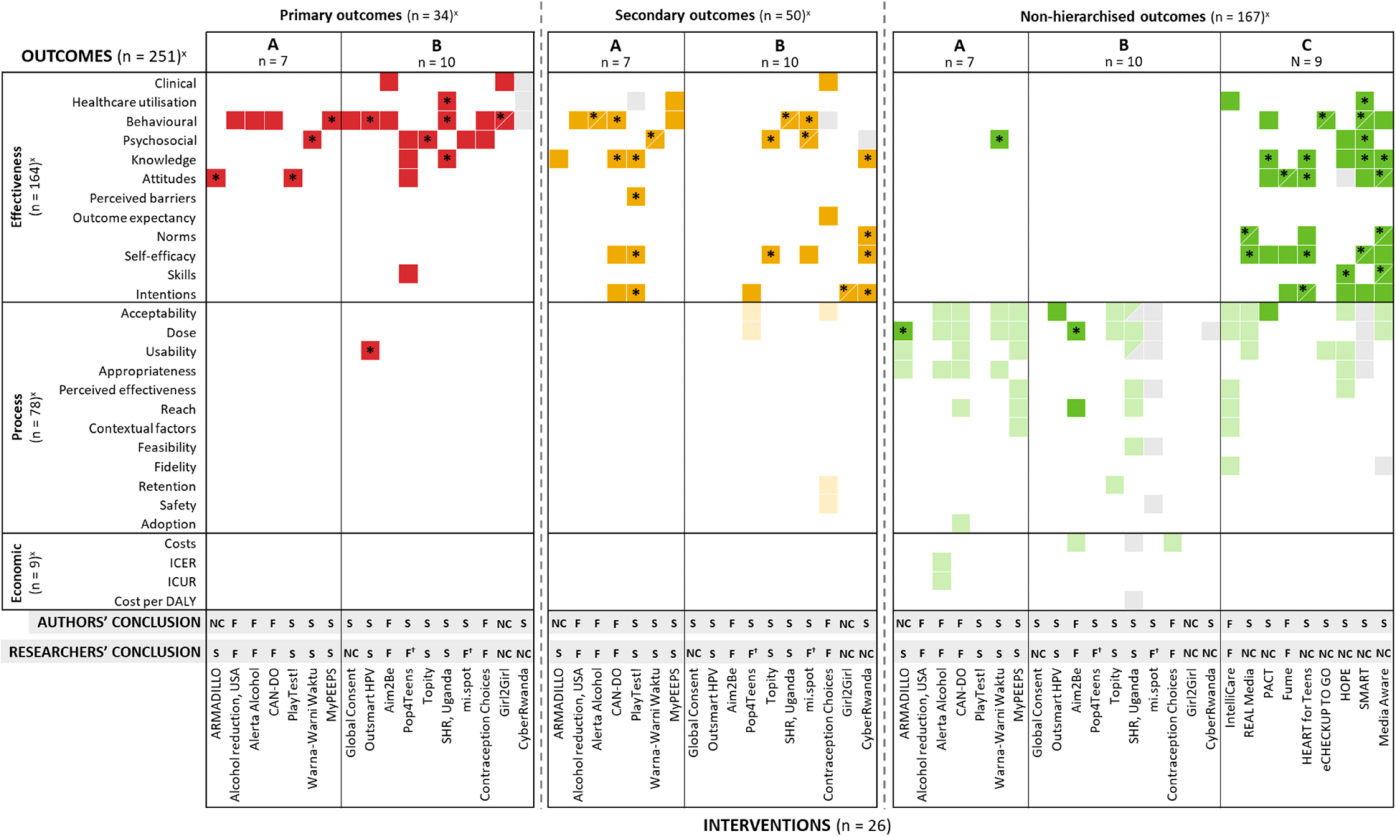
Description of outcome nature, hierarchical position, and statistical results by intervention category, with authors’ and researchers’ conclusions on success ^x^Number of reported outcomes excludes the 42 outcomes mentioned in protocols only. Intervention names are listed at the bottom of the figure. **Intervention categorisation:** A: intervention studies defining a unique primary outcome, with or without additional outcomes. B: intervention studies defining multiple primary outcomes, with or without additional outcomes. C: intervention studies defining multiple non-hierarchised outcomes. Interventions in categories A and B are displayed across the three outcome hierarchical positions—primary, secondary, and non-hierarchised—and therefore appear three times in the figure. **Outcome** **hierarchical** **position:** Red: primary outcomes. Yellow: secondary outcomes. Green: non-hierarchised outcomes. Grey: outcomes in protocols only (*n* = 42), not included in the total outcome count. **Outcome statistical result:** Dark shade with an asterisk (*): statistically significant outcomes (*p* < 0.05). Dark shade without an asterisk: not statistically significant outcomes (*p* ≥ 0.05). Light shade: outcomes not subjected to statistical testing. **Conclusions on intervention success:** S, success; F, failure; NC, non-conclusive; F†, spin (when authors reported success despite all primary outcomes being not statistically significant). **Abbreviations:** ICER, incremental cost-effectiveness ratio; ICUR, incremental cost-utility ratio; DALY, disability-adjusted life years; SRH, sexual and reproductive health. **How to read the figure:** for example, in the ARMADILLO study (category A intervention), the unique effectiveness primary outcome (attitudes) was statistically significant, while the effectiveness secondary outcome (knowledge) was not. Three exploratory (non-hierarchised) process outcomes were measured: intervention dose (statistically significant), usability and appropriateness (both not statistically tested). Both the authors and the researchers concluded that the intervention results were non-conclusive (NC)

Seven interventions (7/26) specified a unique primary outcome, with secondary outcomes and with or without additional non-hierarchised outcomes (Fig. 2, category A); 10 interventions (10/26) were assessed on multiple primary outcomes (category B), with or without secondary or non-hierarchised outcomes, and 9 interventions (9/26) were evaluated using solely multiple non-hierarchised outcomes (category C). None of the interventions using multiple outcomes applied a correction for multiplicity.

### Outcomes

Figure 2 presents the distribution of outcomes by their nature (effectiveness, process, economic), hierarchical position (primary, secondary, non-hierarchised), and statistical results (significant, not significant, not tested) across intervention categories (A, B, C). It also compares the conclusions drawn by authors and researchers regarding intervention success.

A total of 251 reported outcomes were identified across the interventions, including 164 effectiveness outcomes from all 26 interventions, 78 process outcomes from 17 interventions, and 9 economic outcomes from 3 interventions. Of the effectiveness outcomes, 132 measured behaviours or behavioural determinants. An additional 42 outcomes, primarily process ones, were reported only in protocols (shown in grey in Fig. 2) and were not included in the total outcome count. Additional file 6: Tables S4–S5 provide detailed characteristics of outcome hierarchical position and measurement.

#### Interventions evaluated with a unique primary outcome (category A, *n* = 7)

All primary and secondary outcomes were effectiveness-related and statistically tested. Five interventions additionally reported non-hierarchised process or economic outcomes. These outcomes were generally not statistically tested, notably because many were exclusively measured in the intervention group.

#### Interventions evaluated with multiple primary outcomes (category B, *n* = 10)

All primary outcomes were effectiveness-related, except for one usability outcome, and statistically tested. Process outcomes were evaluated as secondary outcomes in only two interventions. Half of the interventions reported additional non-hierarchised outcomes, all process and economic endpoints.

#### Interventions evaluated with multiple non-hierarchised outcomes (category C, *n* = 9)

These interventions predominantly assessed effectiveness outcomes, all statistically tested. Six interventions also reported process outcomes, while none evaluated economic endpoints.

Rationales for outcome hierarchical positions were generally not stated for all interventions (categories A, B, and C).

### Role of outcomes in conclusions

In 8/26 interventions, conclusions were based on all reported outcomes, primarily effectiveness, with one also considering a process outcome (see Additional file 7: Figure S1 for details on outcomes used to determine intervention success). In the remaining 18/26 interventions, conclusions were drawn from a subset of reported outcomes, though the rationale for their selection was not stated.

#### Interventions evaluated with a unique primary outcome (category A, *n* = 7)

Two interventions used all reported outcomes to draw conclusions. All interventions used all reported primary and secondary effectiveness outcomes to conclude, with four incorporating non-hierarchised process outcomes. None included economic outcomes.

#### Interventions evaluated with multiple primary outcomes (category B, *n* = 10)

Four interventions used all reported outcomes to draw conclusions. All interventions included all primary effectiveness outcomes, with four supplementing these with secondary effectiveness outcomes. One intervention also included a secondary process outcome, and three used non-hierarchised process outcomes in their conclusions. None included economic outcomes.

#### Interventions evaluated with multiple non-hierarchised outcomes (category C, *n* = 9)

Two interventions used all reported outcomes to draw conclusions. Six used only effectiveness outcomes, while two included process outcomes. One used all but one effectiveness outcome. None used economic outcomes.

### Authors’ and researchers’ conclusions on intervention success

#### Interventions evaluated with a unique primary outcome (category A, *n* = 7)

The research team agreed with the authors’ conclusions for six interventions (three successful, three unsuccessful; Fig. 2, Additional file 8: Tables S6-S7 for detailed agreement percentages). The remaining intervention was deemed successful by the team based on improvement in the unique primary outcome, while the authors considered it non-conclusive due to secondary outcome results.

#### Interventions evaluated with multiple primary outcomes (category B, *n* = 10)

Agreement was reached for six interventions (three successful, two unsuccessful, one non-conclusive). Two interventions were spin, with authors reporting success despite no statistically significant primary outcomes. The remaining two were classified as non-conclusive by the research team, as only some primary outcomes were statistically significant and no correction for multiplicity was applied.

#### Interventions evaluated with multiple non-hierarchised outcomes (category C, *n* = 9)

Agreement was reached for two unsuccessful interventions. The remaining seven, deemed successful by their authors, were considered non-conclusive by the research team due to statistically conflicting results and lack of multiplicity correction.

### Impact of outcome hierarchical position and number on intervention conclusions

Table 2 illustrates the impact of outcome hierarchical position and number on intervention conclusions for three interventions originally evaluated using (1) a single primary outcome, (2) multiple primary outcomes, and (3) multiple non-hierarchised outcomes. Additional file 9: Table S8 provides additional details on the hierarchical position and result of each outcome for these interventions.

**Table 2:**
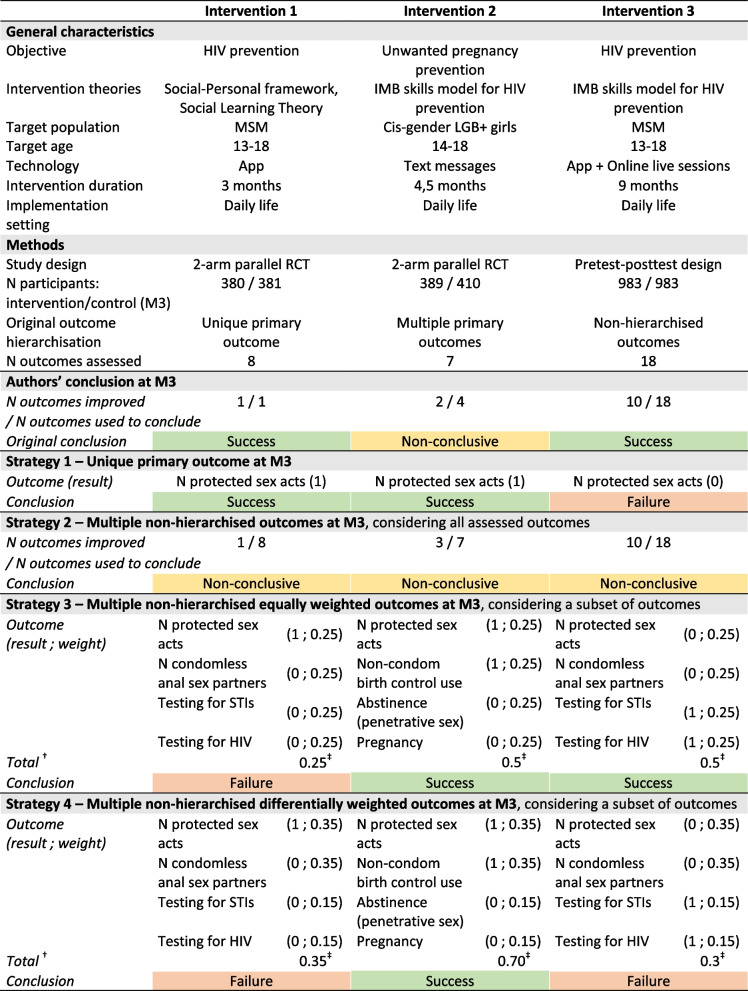
Impact of outcome hierarchical position and number on intervention conclusions (*n* = 3)

^†^Total score calculated by summing the products of each outcome result by its weight; ^‡^arbitrary threshold for intervention success was set at 0.5. **Abbreviations:** *M*, months; *N*, number of; *HIV*, human immunodeficiency virus; *IMB*, Information-Motivation-Behavioural; *MSM*, men who have sex with men; *LGB* + , lesbian, gay, bisexual, and other sexual minority; *App*, mobile application; *RCT*, randomised controlled trial; *STIs*, sexually transmitted infections. **How to read the table:** for example, in intervention 1, under Strategy 1, the unique primary outcome is improved, deeming the intervention successful. Under Strategy 2, eight non-hierarchised outcomes are evaluated, with one improvement, leading to a non-conclusive intervention. Under Strategy 3, four outcomes are equally weighted (0.25 each) with one improved outcome, yielding an intervention score of 0.25, below the success threshold of 0.5, indicating failure. Under Strategy 4, four outcomes are differentially weighted (0.35, 0.35, 0.15, 0.15) with one improved outcome, yielding an intervention score of 0.35, below the success threshold of 0.5, indicating failure

#### Original authors’ conclusions

Intervention 1 was considered successful based on improvement in its unique primary outcome. Intervention 2 was deemed non-conclusive, as only two of its four primary outcomes showed improvement. Intervention 3 was judged successful, although only 10 of the 18 non-hierarchised outcomes showed improvement.

#### Conclusions under alternative strategies

When applying the four predefined strategies, the conclusions on intervention success varied. When success was determined based on the “number of protected sex acts” as the unique primary outcome (Strategy 1), interventions 1 and 2 were successful, while intervention 3 was a failure. Notably, this outcome was measured using three different instruments across the interventions. Under Strategy 2, all interventions were deemed non-conclusive, as none showed improvement across all outcomes. Weighted strategies (Strategies 3 and 4) were applied to four outcomes similarly measured in interventions 1 and 3, and to the four primary outcomes measured in intervention 2. Both weighted strategies classified intervention 2 as successful and intervention 1 as a failure. Intervention 3 was successful under Strategy 3 (equal weighting) but failed under Strategy 4 (differential weighting).

### Risk-of-bias assessment

Most outcomes were self-reported by participants aware of their intervention allocation, leading to some concerns or high risk of bias in outcome measurement for 87% (20/23) of interventions evaluated within a randomised controlled design. Quasi-experimental studies also showed a high risk of outcome measurement bias (3/3). Selective reporting was at low risk of bias in 65% (17/26) of all interventions (see Additional file 10: Figures S2–S3 for the full risk assessment).

## Discussion

### Summary of main findings

This study demonstrates that population health intervention research (PHIR) on adolescents and young adults (AYA) using digital health interventions (DHIs) is evaluated using a wide range of outcomes (effectiveness, process and economic outcomes), as recommended by the MRC guidance [6]. However, intervention success remains predominantly determined by effectiveness outcomes, particularly behaviours and behavioural determinants. While defining primary outcomes simplifies success determination, it requires researchers to prioritise. However, the scientific rationale and processes underpinning this prioritisation are rarely documented, corroborating previous findings [135]. Similarly, authors rarely report their interpretative process for drawing conclusions from multiple outcomes, particularly when results are statistically conflicting and outcomes non-hierarchised. Even in studies with clearly defined primary outcomes, secondary measures frequently influence conclusions, suggesting a more complex interpretation process than initially reported. Finally, this study highlights how methodological decisions on outcome selection and prioritisation substantially shape intervention conclusions.

### Interpretation of results

The reliance on effectiveness outcomes for determining intervention success supports existing evidence of their overemphasis in DHI evaluations [136] and PHIR more broadly [137], despite methodological guidelines advocating for multidimensional assessments [6, 17, 138]. This tendency may arise from the relative ease of interpreting effectiveness outcomes, which are typically assessed through statistical testing with a clear significance threshold, whereas process outcomes are often qualitative and measured exclusively within intervention arms. However, this reflects a key methodological gap: while comprehensive evaluations are needed for informed decision-making, practical guidance on integrating heterogeneous findings across different evaluation phases remains limited (e.g. efficacy, effectiveness, implementation, efficiency, theory-based, and system-based outcomes) [6, 137]. Evidence synthesis is further complicated by inconsistencies in outcome selection and measurement across studies and, while diverse stakeholder perspectives on outcome selection enrich the analysis, they add to this challenge [139]. Thus, the lack of methodological guidance may explain why researchers struggle to articulate their interpretative processes, especially when incorporating non-primary outcomes into conclusions. However, reported processes suggest that authors distinguish intervention effectiveness from intervention success, with success encompassing a broader interpretation of findings across multiple dimensions. Yet, reproducing conclusions is challenging without transparent documentation of interpretative procedures, potentially explaining discrepancies between authors' and researchers' conclusions in this study. As in this study, researchers must rely on published *Methods* sections, which typically limit interpretation to the statistical results of primary or secondary outcomes, most often effectiveness outcomes, whereas authors may draw on a broader range of information, including additional secondary or non-hierarchised outcomes, unpublished data or contextual knowledge when determining success.

The overreliance on effectiveness outcomes may also reflect the prevailing research environment shaped by the dominance of the evidence-based medicine paradigm since the 1990s [140], which emerged as a means to prevent the implementation of untested and sometimes harmful interventions. However, longstanding investment in clinical research has reinforced an evaluation model prioritising randomised controlled trials and hierarchised outcomes, even in fields such as health promotion and population-health intervention research where such designs may not always be suitable. Notably, the requirement to pre-specify a hierarchy of outcomes may overshadow valuable insights from secondary and non-hierarchised outcomes [6, 141–143]. Furthermore, unlike many pharmacological interventions, complex interventions showing positive results in some contexts may not ‘work’ in others, highlighting the importance of complementary studies, such as implementation and transferability research.

Additionally, while effectiveness outcomes may appear fundamental for intervention assessment, this overlooks critical implementation and resource constraints. An effective intervention that cannot be feasibly scaled or is cost-prohibitive ultimately fails to serve public health objectives. This underscores the necessity of integrating implementation considerations into intervention design from inception to reduce research waste [144].

The lack of clarity in outcome selection, measurement, and prioritisation for determining intervention success, particularly when multiple outcomes are involved, presents a significant challenge. Key risks include heterogeneity in outcome definitions, inconsistent measures for assessing similar interventions (e.g. sexual health), and difficulties ensuring result reproducibility. These issues hinder meta-analyses, cross-study comparisons, and interpretation of findings, especially when similar interventions are evaluated in different contexts. In clinical research, the Core Outcome Measures in Effectiveness Trials (COMET) Initiative was established to mitigate outcome heterogeneity within the same clinical context [145]. Given its unique complexities, PHIR could benefit from a similar framework tailored to its needs. Developing such a framework would require engaging diverse stakeholders, including public health researchers, citizens, policymakers, health technology assessment (HTA) bodies, developers, and information technology (IT) specialists (notably for DHIs), and economists. Additionally, outcome selection and prioritisation should align with established theoretical frameworks and the intervention’s theoretical model or be informed by existing evidence from systematic reviews, similar interventions, and contexts [146].

Beyond outcome selection, given that multiple outcomes are necessary to assess the complexity of interventions from different stakeholders’ perspectives, new methodological approaches to outcome combination are needed. Multiple criteria decision analysis (MCDA), widely used in other research fields and increasingly in HTA [34], employs multi-stakeholders qualitative discussions, quantitative weighting, or decision rules to prioritise, rank, or select interventions based on multiple quantitative and qualitative criteria [34, 147]. Similarly, consensus-based approaches could be adapted to synthesise multidimensional information to determine intervention success [148]. Seldom used in PHIR, these methods warrant further exploration and adaptation to integrate the wide range of outcomes measured within complex interventions.

Finally, failure to document outcome selection and prioritisation processes undermines research integrity, as conclusions may be drawn post hoc rather than based on pre-specified measures [149]. To ensure transparency, success criteria should be pre-defined, alongside prioritisation and combination strategies, acknowledging stakeholders’ diverse objectives. While not all measured outcomes will inform final decisions, they may provide valuable insights into unintended effects, mechanisms of actions, contextual influences, and scalability potential [6, 150].

### Future directions

These findings underscore the need for greater transparency in communicating prioritisation criteria and decision-making processes to enhance methodological rigour in assessing the success of population health interventions based on multiple, multidimensional outcomes.

To support these improvements, we propose structuring the governance of research projects through a multidisciplinary steering committee established at the project’s outset, comprising key stakeholders such as academic researchers, funders, implementers, and end-users.

Prior to the study, this committee would define a set of theoretically grounded, valid, reproducible, measurable, and responsive outcomes across at least the effectiveness, process, and economic dimensions. Each stakeholder would prioritise these outcomes according to existing evidence and their preferences and develop a predefined analytical framework that integrates quantitative and qualitative outcomes, accounting for contextual influences to determine intervention success [150]. Where possible, the committee would develop and document a consensus-based analytical plan for combining outcomes and formulating conclusions.

During the study, the committee would monitor project progress, document contextual influences, and identify challenges related to recruitment, data collection, and intervention fidelity.

Upon study completion, the committee would ensure the rigorous application of predefined analytical and interpretative procedures, considering any challenges encountered during implementation. The committee would critically appraise the results—particularly when both beneficial and adverse effects are observed—assess their generalisability in light of contextual factors, and deliver a well-reasoned, context-specific, and consensus-based conclusion regarding intervention success. Additionally, it would outline the risks and benefits of scaling up the intervention and transparently report any deviations from the initial analytical framework.

Even with improved outcome prioritisation and interpretation reporting, statistical challenges related to multiple outcome testing remain, particularly the risk of type I error inflation. While various statistical adjustments exist, their conservatism or underlying assumptions of hypothesis independence may limit their applicability in PHIR [151, 152]. Future research should explore the statistical implications of multiple outcome assessments and the inclusion of outcomes not typically associated with *p*-values to complement theoretical reflections in PHIR.

### Strengths and limitations

This study represents the first systematic methodological examination of how multiple outcomes are interpreted to determine DHI success. By comprehensively reviewing all published materials related to eligible interventions and reporting on each measured outcome, the research offers novel insights into evaluation practices and methodological challenges often overlooked when considering articles individually.

Several limitations must be acknowledged. The research team’s assessment of intervention success was based solely on outcomes reported in peer-reviewed publications. This may be considered a conservative analytical approach that does not account for effect sizes, clinical significance, or partial improvements of overarching behaviours. In practice, authors would typically consider these elements alongside unpublished data, contextual knowledge, and professional expertise when evaluating success. However, the chosen analytical approach was deliberately adopted to underscore the gap that may arise in the conclusion-drawing processes between authors and readers in the absence of transparent interpretation procedures. This conservative methodology was also chosen for our sub-analysis to demonstrate how the analytical approach influences interpretation, rather than to provide definitive assessments of intervention success. The limited sample size and exclusive focus on individual-level DHIs may restrict generalisability, particularly as PHIR often evaluates community-level interventions. However, as the study focuses on methodological decisions rather than the health implications of results, the findings likely reflect key challenges in evaluating multiple outcomes. The proposed methodological improvements are expected to be relevant to other health promotion interventions, especially given that identifying a unique, widely accepted primary outcome in population-wide studies is likely to be even more challenging than in individual-level interventions.

The impact of outcome hierarchical position on conclusions was explored using only effectiveness outcomes, excluding process, economic, qualitative, and non-statistically tested outcomes. While it simplified the demonstration of how methodological choices influence conclusions, this approach did not capture the complexity of integrating quantitative and qualitative outcomes, warranting further investigation.

Finally, this study focused solely on outcomes, while other methodological aspects such as research programme design, study designs, and comparators also influence intervention success determination. Future analysis should explore these to complement existing PHIR literature.

## Conclusions

This study critically examines the methodological processes underpinning outcome selection, analysis, and interpretation in determining the success of complex digital health interventions. Findings reveal that limited transparency in outcome prioritisation and inadequate reporting of interpretative processes may undermine the trustworthiness and reproducibility of conclusions regarding success.

To improve evaluation practices, we propose (1) developing core outcome sets specific to PHIR, (2) collaboratively selecting multidimensional outcomes through a steering committee that accounts for stakeholder preferences and existing theoretical models, (3) exploring MCDA and consensus-driven methods to transparently combine outcomes, with biostatisticians tailoring these methods to PHIR, (4) enhancing methodological reporting at each stage of intervention development and evaluation to improve scientific integrity and reproducibility, and (5) increasing the involvement of PHIR experts in ethics, funding, and evaluation committees to improve recognition of evidence produced in this field.

These methodological proposals aim to ensure robust, transparent evaluations that can inform scalable interventions, ultimately strengthening evidence-based population health.

## Supplementary Information

Additional file 1: Table S1. PRISMA checklist for report. Table S2. PRISMA checklist for abstract.

Additional file 2: Search strategies.

Additional file 3: Data extraction form.

Additional file 4: Table S3. Rationale for weights’ assignment to outcomes.

Additional file 5: List of references included in the review, by intervention.

Additional file 6: Table S4. Outcomes’ characteristics. Table S5. Outcomes’ measurement methods.

Additional file 7: Figure S1. Role of outcomes in conclusions.

Additional file 8: Table S6. Inter-rater agreement on conclusions. Table S7. Inter-rater agreement on conclusions by intervention categorisation.

Additional file 9: Table S8. Outcomes assessment in the sub-analysis.

Additional file 10: Fig. S2. Risk-of-bias assessment (ROB-2) for randomised controlled trials. Fig. S3. Risk-of-bias assessment (ROBINS-I) for non-randomised studies.

## Data Availability

The datasets used and/or analysed during the current study are available from the corresponding author upon reasonable request.

## Abbreviations

AYA: Adolescents and young adults
COMET: Core Outcome Measures in Effectiveness Trials
CONSORT: Consolidated Standards of Reporting Trials
DALY: Disability-adjusted life year
DHI: Digital health intervention
EBM: Evidence-based medicine
HIV: Human immunodeficiency virus
HTA: Health technology assessment
ICER: Incremental cost-effectiveness ratio
ICUR: Incremental cost-utility ratio
IMB: Information-motivation-behavioural
IT: Information technology
LGB +: Lesbian, gay, bisexual, and other sexual minority
MCDA: Multiple criteria decision analysis
MSM: Men who have sex with men
PHIR: Population health intervention research
PRISMA: Preferred Reporting Items for Systematic Reviews and Meta-Analyses
RCT: Randomised controlled trial
SMS: Short message service
SRH: Sexual and reproductive health
STIs: Sexually transmitted infections
UK MRC: United Kingdom Medical Research Council
WHO: World Health Organisation
