# Supplementary material for: A scoping review of outcome selection and accuracy of conclusions in complex digital health interventions for young people (2017–2023): methodological proposals for population health intervention research
Source: BMC Med. 2025 Jul 2;23:400. doi: 10.1186/s12916-025-04245-1 (PMC12224660; doi:10.1186/s12916-025-04245-1)
Supplement: Supplementary file 2 — Additional file 2: Search strategies. [file 12916_2025_4245_MOESM2_ESM.docx]

## Additional File 2. Search strategies

**PubMed**

Search history

| Date | Number of references retrieved | Number of duplicates | Number of references added to screen |
| --- | --- | --- | --- |
| February 2^nd^ 2023 | 1,349 | 0 | 1,349 |
| May 2^nd^ 2023 | 31 | 4 | 27 |
| January 2^nd^ 2024 | 61 | 6 | 55 |
|  | **1,441** | **10** | **1,431** |

Search terms

(("Telemedicine"[MeSH Terms] OR "telemedicine"[Text Word] OR "mhealth"[Text Word] OR "mobile"[Text Word] OR "ehealth"[Text Word] OR "telehealth"[Text Word] OR "Internet"[MeSH Terms] OR "internet"[Text Word] OR "online"[Text Word] OR "web-based"[Text Word] OR "web based"[Text Word] OR "website*"[Text Word] OR "digital"[Text Word] OR "email*"[Text Word] OR "serious game*" [Text Word] OR "computer*"[Text Word] OR "computer-based"[Text Word] OR "Internet-Based Intervention"[MeSH Terms] OR "Mobile Applications"[MeSH Terms] OR "app"[Text Word] OR "apps"[Text Word] OR "Cell Phone"[MeSH Terms] OR "cell phone*"[Text Word] OR "text-messag*"[Text Word] OR "sms"[Text Word] OR "smart phone*"[Text Word] OR "smartphone*"[Text Word] OR "Social Media"[MeSH Terms] OR "social media"[Text Word] OR "social network*"[Text Word] OR "blog*"[Text Word] OR "forum"[Text Word] OR "YouTube"[Text Word] OR "Snapchat"[Text Word] OR "TikTok"[Text Word] OR "Instagram"[Text Word] OR "Twitter"[Text Word] OR "Discord"[Text Word] OR "Facebook"[Text Word] OR "Reddit"[Text Word] OR "Twitch"[Text Word] OR "Tumblr"[Text Word] OR "WhatsApp"[Text Word] OR "Messenger"[Text Word] OR "WeChat"[Text Word] OR "Telegram"[Text Word] OR "MySpace"[Text Word] OR "podcast*"[Text Word] OR "tablet*"[Text Word] OR "newsletter*"[Text Word])

**AND** (("intervention*"[Text Word] OR "action*"[Text Word] OR "program*"[Text Word] OR "initiative*"[Text Word] OR "project*"[Text Word]) AND ("Health Education"[MeSH Terms] OR "health education"[Text Word] OR "education, health"[Text Word] OR "education program*"[Text Word] OR "Health Promotion"[MeSH Terms] OR "health promotion"[Text Word] OR "promotion, health"[Text Word] OR "promotion program*"[Text Word] OR "Health Communication"[MeSH Terms] OR "health communication*"[Text Word] OR "Primary Prevention"[MeSH Terms] OR "primary prevention*"[Text Word] OR "primordial prevention*"[Text Word] OR "Preventive Health Services"[MeSH Terms] OR "preventive health service*" [Text Word]))

**AND** ("Adolescent"[MeSH Terms] OR "adolescent*"[Text Word] OR "adolescence"[Text Word] OR "youth"[Text Word] OR "youths"[Text Word] OR "teen*"[Text Word] OR "Young Adult"[MeSH Terms] OR "young adult*"[Text Word] OR "young people"[Text Word] OR "young person*"[Text Word] OR "AYA"[Text Word])

**AND** ("evaluat*"[Text Word] OR "outcome*"[Text Word] OR "evaluability"[Text Word] OR "feasibility"[Text Word] OR "acceptability"[Text Word] OR "adherence"[Text Word] OR "engagement"[Text Word] OR "usability"[Text Word] OR "attractiveness"[Text Word] OR "efficacy"[Text Word] OR "effectiveness"[Text Word] OR "impact"[Text Word] OR "side-effect*"[Text Word] OR "side effect*"[Text Word] OR "process evaluation"[Text Word] OR "process result*"[Text Word] OR "implementation"[Text Word] OR "fidelity"[Text Word] OR "mechanism of change"[Text Word] OR "mechanisms of change"[Text Word] OR "economic evaluation"[Text Word] OR "efficiency"[Text Word] OR "cost-effectiveness"[Text Word] OR "cost effectiveness"[Text Word] OR "cost-benefit"[Text Word] OR "cost benefit"[Text Word] OR "cost-utility"[Text Word] OR "cost utility"[Text Word] OR "transferability"[Text Word])

**AND** (("mixed method*"[All Fields] OR "mixed-method*" [All Fields] OR "multimethod*"[All Fields] OR "multi-method*"[All Fields] OR "combined method*"[All Fields] OR "combined approach*"[All Fields] OR "combined process*"[All Fields] OR "composite approach*"[All Fields]) OR (("efficacy"[All Fields] OR "effectiveness"[All Fields] OR "impact"[All Fields] OR "side-effect*"[All Fields] OR "side effect*"[All Fields]) AND ("feasibility"[All Fields] OR "acceptability"[All Fields] OR "adherence"[All Fields] OR "engagement"[All Fields] OR "usability"[All Fields] OR "attractiveness"[All Fields] OR "process evaluation"[All Fields] OR "process result*"[All Fields] OR "implementation"[All Fields] OR "fidelity"[All Fields] OR "mechanism of change"[All Fields] OR "mechanisms of change"[All Fields] OR "economic"[All Fields] OR "efficiency"[All Fields] OR "cost*"[All Fields])) OR ("quantitativ*"[All Fields] AND "qualitativ*"[All Fields])))

**NOT** ("Secondary Prevention"[MeSH Terms] OR "secondary prevention*"[Text Word] OR "Tertiary Prevention"[MeSH Terms] OR "tertiary prevention*"[Text Word])

**NOT** ("RNA, Messenger"[MeSH Terms] OR "messenger RNA"[Text Word])

************************

**CINAHL**

Search history

| Date | Number of references retrieved | Number of duplicates | Number of references added to screen |
| --- | --- | --- | --- |
| February 2^nd^ 2023 | 289 | 193 | 96 |
| May 2^nd^ 2023 | 12 | 4 | 8 |
| January 2^nd^ 2024 | 26 | 8 | 18 |
|  | **327** | **205** | **122** |

Search terms

(("telemedicine" OR "mhealth" OR "mobile" OR "ehealth" OR "telehealth" OR "internet" OR "online" OR "web-based" OR "web based" OR "website" OR "digital" OR "email" OR "serious game" OR "computer" OR "computer-based" OR "internet-based intervention" OR "mobile application" OR "app" OR "apps" OR "cell phone" OR "text-messaging" OR "text-message" OR "sms" OR "smart phone" OR "smartphone" OR "social media" OR "social network" OR "blog" OR "forum" OR "YouTube" OR "Snapchat" OR "TikTok" OR "Instagram" OR "Twitter" OR "Discord" OR "Facebook" OR "Reddit" OR "Twitch" OR "Tumblr" OR "WhatsApp" OR "Messenger" OR "WeChat" OR "Telegram" OR "MySpace" OR "podcast" OR "tablet" OR "newsletter")

**AND** (("intervention" OR "action" OR "program" OR "initiative" OR "project") AND ("health education" OR "education program" OR "health promotion" OR "promotion program" OR "health communication" OR "primary prevention" OR "primordial prevention" OR "preventive health services"))

**AND** ("adolescent" OR "adolescence" OR "youth" OR "youths" OR "teen" OR "young adult" OR "young people" OR "young person" OR "AYA")

**AND** ("evaluation" OR "outcome" OR "evaluability" OR "feasibility" OR "acceptability" OR "adherence" OR "engagement" OR "usability" OR "attractiveness" OR "efficacy" OR "effectiveness" OR "impact" OR "side-effect" OR "side effect" OR "process evaluation" OR "process results" OR "implementation" OR "fidelity" OR "mechanism of change" OR "mechanisms of change" OR "economic evaluation" OR "efficiency" OR "cost-effectiveness" OR "cost effectiveness" OR "cost-benefit" OR "cost benefit" OR "cost-utility" OR "cost utility" OR "transferability")

**AND** (("mixed method" OR "mixed-method" OR "multimethod" OR "multi-method" OR "combined method" OR "combined approach" OR "combined process" OR "composite approach") OR (("efficacy" OR "effectiveness" OR "impact" OR "side-effect" OR "side effect") AND ("feasibility" OR "acceptability" OR "adherence" OR "engagement" OR "usability" OR "attractiveness" OR "process evaluation" OR "process result" OR "process results" OR "implementation" OR "fidelity" OR "mechanism of change" OR "mechanisms of change" OR "economic" OR "efficiency" OR "cost")) OR ("quantitative" AND "qualitative")))

**NOT** ("secondary prevention" OR "tertiary prevention")

**NOT** ("messenger RNA")

************************

**EMBASE**

Search history

| Date | Number of references retrieved | Number of duplicates | Number of references added to screen |
| --- | --- | --- | --- |
| February 2^nd^ 2023 | 2,344 | 662 | 1,682 |
| May 2^nd^ 2023 | 99 | 16 | 83 |
| January 2^nd^ 2024 | 452 | 226 | 226 |
|  | **2,895** | **904** | **1,991** |

Search terms

('telemedicine'/exp OR 'telemedicine' OR 'mhealth'/exp OR 'mhealth' OR 'mobile' OR 'ehealth'/exp OR 'ehealth' OR 'telehealth'/exp OR 'telehealth' OR 'internet'/exp OR 'internet' OR 'online'/exp OR 'online' OR 'web-based' OR 'web based' OR 'website'/exp OR 'website*' OR 'digital' OR 'email'/exp OR 'email' OR 'serious game'/exp OR 'serious game*' OR 'computer'/exp OR 'computer*' OR 'computer-based' OR 'internet-based intervention'/exp OR 'internet-based intervention*' OR 'mobile application'/exp OR 'mobile application*' OR 'app' OR 'apps' OR 'cell phone'/exp OR 'cell phone*' OR 'text-messaging'/exp OR 'text-messag*' OR 'text-message' OR 'sms' OR 'smart phone'/exp OR 'smart phone*' OR 'smartphone'/exp OR 'smartphone*' OR 'social media'/exp OR 'social media' OR 'social network'/exp OR 'social network*' OR 'blog'/exp OR 'blog*' OR 'forum' OR 'youtube'/exp OR 'youtube' OR 'snapchat'/exp OR 'snapchat' OR 'tiktok'/exp OR 'tiktok' OR 'instagram'/exp OR 'instagram' OR 'twitter'/exp OR 'twitter' OR 'discord' OR 'facebook'/exp OR 'facebook' OR 'reddit'/exp OR 'reddit' OR 'twitch'/exp OR 'twitch' OR 'tumblr'/exp OR 'tumblr' OR 'whatsapp'/exp OR 'whatsapp' OR 'messenger' OR 'wechat'/exp OR 'wechat' OR 'telegram' OR 'myspace'/exp OR 'myspace' OR 'podcast'/exp OR 'podcast*' OR 'tablet'/exp OR 'tablet*' OR 'newsletter'/exp OR 'newsletter*')

**AND** (('intervention'/exp OR 'intervention*' OR 'action'/exp OR 'action*' OR 'program'/exp OR 'program*' OR 'initiative*' OR 'project*') AND ('health education'/exp OR 'health education' OR 'education program'/exp OR 'education program*' OR 'health promotion'/exp OR 'health promotion' OR 'promotion program*' OR 'health communication'/exp OR 'health communication' OR 'primary prevention'/exp OR 'primary prevention' OR 'primordial prevention'/exp OR 'primordial prevention' OR 'preventive health services'/exp OR 'prevention health service*'))

**AND** ('adolescent'/exp OR 'adolescent*' OR 'adolescence'/exp OR 'adolescence' OR 'youth'/exp OR 'youth' OR 'youths' OR 'teen*' OR 'young adult'/exp OR 'young adult*' OR 'young people'/exp OR 'young people' OR 'young person*' OR 'aya')

**AND** ('evaluation'/exp OR 'evaluat*' OR 'outcome'/exp OR 'outcome*' OR 'evaluability' OR 'feasibility'/exp OR 'feasibility' OR 'acceptability'/exp OR 'acceptability' OR 'adherence'/exp OR 'adherence' OR 'engagement'/exp OR 'engagement' OR 'usability'/exp OR 'usability' OR 'attractiveness'/exp OR 'attractiveness' OR 'efficacy'/exp OR 'efficacy' OR 'effectiveness' OR 'impact'/exp OR 'impact' OR 'side-effect'/exp OR 'side-effect*' OR 'side effect'/exp OR 'side effect*' OR 'process evaluation'/exp OR 'process evaluation' OR 'process results'/exp OR 'process result*' OR 'implementation'/exp OR 'implementation' OR 'fidelity'/exp OR 'fidelity' OR 'mechanism of change' OR 'mechanisms of change' OR 'economic evaluation'/exp OR 'economic evaluation' OR 'efficiency'/exp OR 'efficiency' OR 'cost-effectiveness'/exp OR 'cost-effectiveness' OR 'cost effectiveness'/exp OR 'cost effectiveness' OR 'cost-benefit'/exp OR 'cost-benefit' OR 'cost benefit'/exp OR 'cost benefit' OR 'cost-utility'/exp OR 'cost-utility' OR 'cost utility'/exp OR 'cost utility' OR 'transferability'/exp OR 'transferability')

**AND** ('mixed method'/exp OR 'mixed method*' OR 'mixed-method*' OR 'multimethod*' OR 'multi-method*' OR 'combined method*' OR 'combined approach*' OR 'combined process' OR 'composite approach' OR (('efficacy'/exp OR 'efficacy' OR 'effectiveness' OR 'impact'/exp OR 'impact' OR 'side-effect'/exp OR 'side-effect*' OR 'side effect'/exp OR 'side effect*') AND ('feasibility'/exp OR 'feasibility' OR 'acceptability'/exp OR 'acceptability' OR 'adherence'/exp OR 'adherence' OR 'engagement'/exp OR 'engagement' OR 'usability'/exp OR 'usability' OR 'attractiveness'/exp OR 'attractiveness' OR 'process evaluation'/exp OR 'process evaluation' OR 'process results'/exp OR 'process result*' OR 'implementation'/exp OR 'implementation' OR 'fidelity'/exp OR 'fidelity' OR 'mechanism of change' OR 'mechanisms of change' OR 'economic' OR 'efficiency'/exp OR 'efficiency' OR 'cost'/exp OR 'cost')) OR ('quantitative' AND ('qualitative'/exp OR 'qualitative')))

**NOT** ('secondary prevention'/exp OR 'secondary prevention' OR 'tertiary prevention'/exp OR 'tertiary prevention')

**NOT** ('messenger rna'/exp OR 'messenger rna')

************************

**PsycINFO**

Search history

| Date | Number of references retrieved | Number of duplicates | Number of references added to screen |
| --- | --- | --- | --- |
| February 2^nd^ 2023 | 1,112 | 109 | 1,003 |
| May 2^nd^ 2023 | 60 | 33 | 27 |
| January 2^nd^ 2024 | 165 | 10 | 155 |
|  | **1,337** | **152** | **1,185** |

Search terms

(e-health or ehealth or digital health or telemedicine or telehealth or internet-based intervention or mhealth or mobile health or m-health or mobile app or mobile application or smartphone application or app or apps or internet or web-based interventions' or 'e-health' or 'internet-based interventions or website or smartphone or online email serious games or video games or digital games or computer or computer-based or computer-based intervention or cellphones or cell phones or sms or text messaging or short message or text messages or smartphone or smart phone or blogs or social media or social network or podcast or podcasts or podcasting or tablet or newsletter

**AND**

initiatives or programs or strategy or intervention or project or action

**AND**

health education or health promotion or promotion or promote or promoting or health communication or communication, health or communications, health or health communications or primary prevention or primordial prevention or preventive health services

**AND**

adolescents or teenagers or young adults or teen or youth or adolescence or young people or young person or youths or teens

**AND**

evaluation methods or evaluation process or evaluation or efficacy or effectiveness or impact or benefits or outcomes or success or efficiency or outcome measures or evaluability or feasibility or acceptability or adherence or engagement or usability or user experience or attractiveness or side effects or adverse effects or process evaluation or implementation evaluation or fidelity of implementation or implementation fidelity or mechanisms of change or economic evaluation or cost economic or economic analysis or cost-effectiveness or cost-benefit or cost-consequence or cost-utility or transferability

**AND**

mixed methods or 'qualitative' and 'quantitative'

**NOT** secondary prevention or secondary disease prevention or tertiary prevention or tertiary education

**NOT** messenger rna)

************************

**ClinicalTrials.gov**

Search history

| Date | Number of references retrieved | Number of duplicates | Number of references added to screen |
| --- | --- | --- | --- |
| January 2^nd^ 2024 | **690** | **31** | **659** |

Search terms

("ehealth" OR "web-based" OR "online" OR "internet" OR "digital" OR "mobile") AND ("promotion" OR "prevention") AND ("adolescent" OR "youth") AND ("evaluation" OR "outcome" OR "feasibility" OR "effectiveness" OR "process" OR "economic")

With filters:

• 10 Years to 25 Years old

• Interventional studies
