# Supplementary material for: A scoping review of outcome selection and accuracy of conclusions in complex digital health interventions for young people (2017–2023): methodological proposals for population health intervention research
Source: BMC Med. 2025 Jul 2;23:400. doi: 10.1186/s12916-025-04245-1 (PMC12224660; doi:10.1186/s12916-025-04245-1)
Supplement: Supplementary file 3 — Additional file 3: Data extraction form. [file 12916_2025_4245_MOESM3_ESM.docx]

## Additional File 3. Data extraction form

| **CHARACTERISTICS OF THE INTERVENTION** |
| --- |
| **Name:** *Text field*  **Description:** *Text field*  **Start date (including recruitment):** mm/yyyy  **End date:** mm/yyyy  **Target population:**  □ General AYA (Adolescents and Young Adults) population  □ Specific AYA population. **Specify:** *Text field*  **Age targeted by the intervention.** Lower limit: \|_\|\|_\| years. Upper limit: \|_\|\|_\| years.  **Health topic(s):**  □ Nutrition and physical activity □ Tobacco □ Alcohol  □ Gambling addiction □ Illicit substance uses □ Sexual and reproductive health  □ Vaccination and vaccine-preventable diseases □ Mental health  □ Other: *Text field*  **Planned dose/usage of the intervention:** □ Single exposure □ Repeated exposure □ NR  **If repeated exposure, what is the duration of the exposure?** *Text field*  **If repeated exposure, what is the frequency of the exposure?**  □ As desired by the user □ Other: *Text field* □ NR  **Does the content of the intervention received vary based on participant characteristics?**  □ No, no personalisation □ Yes, for groups of participants with similar “risks”  □ Yes, individual personalisation (different version of the intervention for each participant)  □ NR  **Method(s) used to develop the intervention:**  □ Existing intervention  □ Existing intervention transferred and/adapted to a new population or context  □ Nex intervention based on theory  □ New intervention based on practice or public policies  □ Other: *Text field* □ NR  **Digital technology(ies) used in the intervention:**  **Platform dedicated to the intervention (website, app):** □ Yes □ No □ NR If yes, specify the digital components used: *Text field* (e.g., videos, instant discussions) **Social media (blog, forum, social network, podcast platform):** □ Yes □ No □ NR If yes, specify the social media and components used: *Text field* (e.g., social network name such as Twitter, Snapchat, etc., forums, videos, information messages) **Serious game:** □ Yes □ No □ NR If yes, specify: □ Online/multiplayer game □ Offline/local game **Messaging service (SMS, audio, email, newsletter):** □ Yes □ No □ NR If yes, specify: *Text field* (e.g., SMS, interactive audio) **Telephone call or videoconference service:** □ Yes □ No □ NR If yes, specify: *Text field* (e.g., phone call, Zoom platform)  **Mobile device:** □ Yes □ No □ NR If yes, specify: *Text field* (e.g., tracker, sensor) **Other:** □ Yes □ No If yes, specify: Text field  **Number of intervention components described by the author(s):** \|_\|\|_\| □ NR **Intervention component(s) described by the author(s):** □ Components for disseminating information, education, and promoting specific behaviours (e.g., videos, text messages (SMS, emails, articles, tips), audio messages, graphics, FAQs, etc.) [Passive engagement of the participant] □ Components for standardised interaction between the participant and the digital platform (e.g., interactive voice response, personal goal setting, interactive decision tools, quizzes, etc.) [Active engagement with the platform] □ Components for direct interaction between participants and their peers (live sessions, discussion forums/testimonials, Q&A, phone call, video conference, etc.) [Active engagement with peers through the platform] □ Components for direct interaction between participants and health/promotion professionals (live sessions, discussion forums, Q&A, phone call, video conference, etc.) [Active engagement with professionals through the platform] □ Digital sensor or tracker for data collection/monitoring [No direct engagement of the participant] □ Other: *Text field*  □ NR |
| **CHARACTERISTICS OF PARTICIPANTS RECEIVING THE INTERVENTION** |
| **Number of participants receiving the intervention in the effectiveness evaluation:** n = \|_\|\|_\|\|_\|\|_\|\|_\|  **How were these participants selected to receive the intervention in the effectiveness evaluation?**  □ Randomisation □ Volunteering □ Other: *Text field* □ NR |

| **GENERAL INFORMATION ON OUTCOMES** |
| --- |
| **Is there a published or registered protocol for this study?** □ Yes □ No  **If yes, total number of outcomes defined (according to the protocol):** \|_\|\|_\|  **Total number of outcomes defined (according to the Methods):** \|_\|\|_\|  **Notes if differences exist between the protocol and the study:** *Text field*  **Hierarchy between primary and secondary outcomes?** □ Yes □ No  **If yes, total number of secondary outcomes:** \|_\|\|_\|  **Total number of quantitative outcomes measured:** \|_\|\|_\|  **Total number of qualitative outcomes measured:** \|_\|\|_\| |
| **QUANTITATIVE OUTCOME** |
| *This section is filled for each quantitative outcome reported for the evaluation of the intervention.*  **Outcome name:** *Text field*  **Hierarchical position:** □ No hierarchy in this study □ Primary □ Secondary □ Mediator □ Moderator  **Dimension assessed by the outcome:** □ Effectiveness □ Process □ Economic □ Other: *Text field*  **Domain assessed by the outcome:** *Text field* (e.g. behaviour, attitudes, acceptability, etc.)  **Sample size for which the outcome is measured (NR, 9999):** n = \|_\|\|_\|\|_\|\|_\|\|_\|\|_\|  **Data collection instrument:**  □ Medical device (e.g., scales, blood pressure monitor, thermometer, etc.)  □ Human samples (e.g., blood test data, etc.)  □ Digital sensor or device  □ Questionnaire  □ Rating scale (e.g., Numeric Rating Scale, Verbal Rating Scale, Visual Analogue Scale, Likert, Graphic Rating Scale, Descriptive Graphic Rating Scale)  □ Digital technology used for intervention delivery (e.g., log in details)  □ Other: *Text field*  **If questionnaire or scale, is it a subjective measure?** □ Yes □ No □ NR  **If yes, is the instrument validated?** □ Yes □ Partially □ No □ NR  **If yes or partially, name of the instrument (+ abbreviation):** *Text field*  **Who collected the data?**  □ Youth (self-administered) □ Parent/guardian of youth □ Healthcare professional  □ Research professional □ Passive collective (sensor/device, digital supports)  □ Other: *Text field*  **How many times is the outcome measured in the study?** \|_\|\|_\|  **When?** □ Pre-intervention/Baseline □ During the intervention  □ At the end of the intervention/endline □ After the end of the intervention/follow-up  **If follow-up measurements, which follow-up times?** *Text field (e.g. M3, M9, M12)*  **What is the main time point of the analysis?** *Text field (e.g. M3, M9, M12)*  **Is the outcome statistically tested at the main time point of the analysis?** □ Yes □ No □ NR  **Is yes, what is the result?** □ Improved (p < 0.05) □ Unimproved (p >= 0.05) □ NR  **Is this outcome addressed by the authors in the Discussion section of the article concerning the intervention's success?** □ Yes □ No |
| **QUALITATIVE OUTCOME** |
| *This section is filled for each qualitative outcome reported for the evaluation of the intervention.*  **Outcome name:** *Text field*  **Hierarchical position:** □ No hierarchy in this study □ Primary □ Secondary □ Mediator □ Moderator  **Dimension assessed by the outcome:** □ Effectiveness □ Process □ Economic □ Other: *Text field*  **Domain assessed by the outcome:** *Text field* (e.g. behaviour, attitudes, acceptability, etc.)  **Sample size for which the outcome is measured (NR, 9999):** n = \|_\|\|_\|\|_\|\|_\|\|_\|\|_\|  **Data collection instrument:** □ Focus group(s) □ Interview(s) □ Observation(s) □ Document analysis  □ Open-ended questionnaire field/text box or written comment  □ Workshop(s) □ Other: *Text field*  Outcome analysis methods: □ Discourse analysis □ Content analysis □ Thematic analysis  □ Narrative analysis □ Case study □ Grounded theory  □ Other: *Text field*  **Who collected the data?**  □ Youth (self-administered) □ Parent/guardian of youth □ Healthcare professional  □ Research professional □ Passive collective (sensor/device, digital supports)  □ Other: *Text field*  **How many times is the outcome measured in the study?** \|_\|\|_\|  **When?** □ Pre-intervention/Baseline □ During the intervention  □ At the end of the intervention/endline □ After the end of the intervention/follow-up  **If follow-up measurements, which follow-up times?** *Text field (e.g. M3, M9, M12)*  **What is the main time point of the analysis?** *Text field (e.g. M3, M9, M12)*  **Is the outcome statistically tested at the main time point of the analysis?** □ Yes □ No □ NR  **Is yes, what is the result?** □ Improved (p < 0.05) □ Unimproved (p >= 0.05) □ NR  **Is this outcome addressed by the authors in the Discussion section of the article concerning the intervention's success?** □ Yes □ No |

Conclusions on intervention success according to the authors were recorded in *Text Fields*.
