# Supplementary material for: A scoping review of outcome selection and accuracy of conclusions in complex digital health interventions for young people (2017–2023): methodological proposals for population health intervention research
Source: BMC Med. 2025 Jul 2;23:400. doi: 10.1186/s12916-025-04245-1 (PMC12224660; doi:10.1186/s12916-025-04245-1)
Supplement: Supplementary file 4 — Additional file 4: Table S3. Rationale for weights’ assignment to outcomes. [file 12916_2025_4245_MOESM4_ESM.docx]

## Additional File 4. Rationale for weight attribution in the simulated case

**Table S3. Rationale for differentially assigned weights in assessing the impact of outcome hierarchical position and number on intervention conclusions**

| **Outcomes** | **Assigned weights (0-1)** | **Rationale** |
| --- | --- | --- |
| **Intervention 1** | | |
| Number of condomless anal sex acts | 0.35 | Represents directly at-risk behaviour. |
| Number of condomless anal sex partners | 0.35 | Represents directly at-risk behaviour. |
| Testing for HIV | 0.15 | Behaviour that indirectly affects the risk of infection or illness is given a smaller weight compared to directly at-risk behaviours. |
| Testing for STIs | 0.15 | Behaviour that indirectly affects the risk of infection or illness is given a smaller weight compared to directly at-risk behaviours. |
| **Intervention 2** | | |
| Pregnancy since program enrolment | 0.15 | A clinical outcome that is unlikely to be measured within three months post-intervention is assigned a smaller weight. |
| Number of condom-protected sex acts | 0.35 | Represents directly at-risk behaviour. |
| Current use of birth control other than condom | 0.35 | Represents directly at-risk behaviour. |
| Abstinence from penile-vaginal sex | 0.15 | Behaviour considered challenging to achieve is assigned a smaller weight. |
| **Intervention 3** | | |
| Number of condomless anal sex acts | 0.35 | Represents directly at-risk behaviour. |
| Number of condomless anal sex partners | 0.35 | Represents directly at-risk behaviour. |
| Testing for HIV | 0.15 | Behaviour that indirectly affects the risk of infection or illness is given a smaller weight compared to directly at-risk behaviours. |
| Testing for STIs | 0.15 | Behaviour that indirectly affects the risk of infection or illness is given a smaller weight compared to directly at-risk behaviours. |

All outcomes were self-reported by the participants.

Weights were also assigned considering existing literature on sexual and reproductive health interventions for young people: (35) Desrosiers A, Betancourt T, Kergoat Y, Servilli C, Saye L, Kobeissi L. A systematic review of sexual and reproductive health interventions for young people in humanitarian and lower-and-middle-income country settings. *BMC Public Health*. 2020;20-1. <https://doi.org/10.1186/s12889-020-08818-y>.
