# Supplementary material for: A scoping review of outcome selection and accuracy of conclusions in complex digital health interventions for young people (2017–2023): methodological proposals for population health intervention research
Source: BMC Med. 2025 Jul 2;23:400. doi: 10.1186/s12916-025-04245-1 (PMC12224660; doi:10.1186/s12916-025-04245-1)
Supplement: Supplementary file 5 — Additional file 5: List of references included in the review, by intervention. [file 12916_2025_4245_MOESM5_ESM.docx]

## Additional File 5. List of references included in the review, by intervention (intervention name, country of implementation)

- ***Sexual and Reproductive Health (SHR) intervention, Uganda***

[36] Nuwamanya E, Nuwasiima A, Babigumira JU, Asiimwe FT, Lubinga SJ, Babigumira JB. Study protocol: using a mobile phone-based application to increase awareness and uptake of sexual and reproductive health services among the youth in Uganda. A randomized controlled trial. *Reprod Health.* 2018;15:216. <https://doi.org/10.1186/s12978-018-0642-0>.

[37] Nuwamanya E, Nalwanga R, Nuwasiima A, Babigumira JU, Asiimwe FT, Babigumira JB, et al. Effectiveness of a mobile phone application to increase access to sexual and reproductive health information, goods, and services among university students in Uganda: a randomized controlled trial. *Contracept Reprod Med.* 2020;5:31. <https://doi.org/10.1186/s40834-020-00134-5>.

[38] Nalwanga R, Nuwamanya E, Nuwasiima A, Babigumira JU, Asiimwe FT, Babigumira JB. Utilization of a mobile phone application to increase access to sexual and reproductive health information, goods, and services among university students in Uganda. *Reprod Health.* 2021;18:95. <https://doi.org/10.1186/s12978-020-01037-z>.

- ***Alcohol reduction intervention, United States of America***

[39] Riordan BC, Conner TS, Flett JA, Scarf D. A Brief Orientation Week Ecological Momentary Intervention to Reduce University Student Alcohol Consumption. *J Stud Alcohol Drugs.* 2015;76. <https://doi.org/10.15288/jsad.2015.76.525>.

[40] Riordan BC, Conner TS, Flett JA, Scarf D. A text message intervention to reduce first year university students’ alcohol use: A pilot experimental study. *Digit Health* 2017;3:2055207617707627. <https://doi.org/10.1177/2055207617707627>.

[41] Riordan BC, Moradi S, Carey KB, Conner TS, Jang K, Reid KE, et al. Effectiveness of a Combined Web-Based and Ecological Momentary Intervention for Incoming First-Year University Students: Protocol for a 3-Arm Randomized Controlled Trial. *JMIR Res Protoc.* 2018;7:e10164. <https://doi.org/10.2196/10164>.

[42] Riordan BC, Winter T, Carey KB, Conner TS, Moradi S, Jang K, et al. A combined web based intervention and ecological momentary intervention for reducing alcohol use among incoming first-year university students: Results from a three-arm randomised controlled trial. *Addict Behav.* 2023;136:107471. <https://doi.org/10.1016/j.addbeh.2022.107471>.

- ***Aim2Be, Canada***

[43] Piatkowski C, Faulkner GE, Guhn M, Mâsse LC. User Characteristics and Parenting Practices Associated with Adolescents’ Initial Use of a Lifestyle Behavior Modification Intervention. *Child Obes.* 2020;16:367–78. <https://doi.org/10.1089/chi.2020.0035>.

[44] Lin Y, Mâsse LC. A look at engagement proFiles and behavior change: A proFile analysis examining engagement with the Aim2Be lifestyle behavior modification app for teens and their families. *Prev Med Rep.* 2021;24:101565. <https://doi.org/10.1016/j.pmedr.2021.101565>.

[45] Mâsse LC, Vlaar J, Macdonald J, Bradbury J, Warshawski T, Buckler EJ, et al. Aim2Be mHealth intervention for children with overweight and obesity: study protocol for a randomized controlled trial. *Trials.* 2020;21:132. <https://doi.org/10.1186/s13063-020-4080-2>.

[46] Tugault-Lafleur CN, De-Jongh González O, Macdonald J, Bradbury J, Warshawski T, Ball GDC, et al. Efficacy of the Aim2Be Intervention in Changing Lifestyle Behaviors Among Adolescents With Overweight and Obesity: Randomized Controlled Trial. *J Med Internet Res.* 2023;25:e38545. <https://doi.org/10.2196/38545>.

[47] De-Jongh González O, Tugault-Lafleur CN, Buckler EJ, Hamilton J, Ho J, Buchholz A, et al. The Aim2Be mHealth Intervention for Children With Overweight or Obesity and Their Parents: Person-Centered Analyses to Uncover Digital Phenotypes. *J Med Internet Res.* 2022;24:e35285. <https://doi.org/10.2196/35285>.

[48] Deslippe AL, González OD-J, Buckler EJ, Ball GDC, Ho J, Bucholz A, et al. Do Individual Characteristics and Social Support Increase Children’s Use of an MHealth Intervention? Findings from the Evaluation of a Behavior Change MHealth App, Aim2Be. *Child Obes.* 2023. <https://doi.org/10.1089/chi.2022.0055>.

[49] Buckler EJ, González OD-J, Ball GDC, Hamilton J, Ho J, Morrison KM, et al. Recruiting families using social media versus pediatric obesity clinics: A secondary analysis of the Aim2Be RCT. *Contemp Clin Trials*. 2023;133:107322. <https://doi.org/10.1016/j.cct.2023.107322>.

- ***Alerta Alcohol, Spain***

[50] Lima-Serrano M, Martínez-Montilla JM, Lima-Rodríguez JS, Mercken L, de Vries H. Design, implementation and evaluation of a web-based computer-tailored intervention to prevent binge drinking in adolescents: study protocol. *BMC Public Health*. 2018;18:449. <https://doi.org/10.1186/s12889-018-5346-4>.

[51] Vargas-Martínez AM, Trapero-Bertran M, Lima-Serrano M, Anokye N, Pokhrel S, Mora T. Measuring the effects on quality of life and alcohol consumption of a program to reduce binge drinking in Spanish adolescents. *Drug Alcohol Depend*. 2019;205:107597. <https://doi.org/10.1016/j.drugalcdep.2019.107597>.

[52] Martinez-Montilla JM, Mercken L, de Vries H, Candel M, Lima-Rodríguez JS, Lima-Serrano M. A Web-Based, Computer-Tailored Intervention to Reduce Alcohol Consumption and Binge Drinking Among Spanish Adolescents: Cluster Randomized Controlled Trial. *J Med Internet Res.* 2020;22:e15438. <https://doi.org/10.2196/15438>.

[53] Vargas-Martínez AM, Lima-Serrano M, Trapero-Bertran M. Cost-effectiveness and cost-utility analyses of a web-based computer-tailored intervention for prevention of binge drinking among Spanish adolescents. *Alcohol Clin Exp Res.* 2023;47:319–35. <https://doi.org/10.1111/acer.14990>.

- ***ARMADILLO, Kenya and Peru***

[54] Gonsalves L, L’Engle KL, Tamrat T, Plourde KF, Mangone ER, Agarwal S, et al. Adolescent/Youth Reproductive Mobile Access and Delivery Initiative for Love and Life Outcomes (ARMADILLO) Study: formative protocol for mHealth platform development and piloting. *Reprod Health*. 2015;12:67. <https://doi.org/10.1186/s12978-015-0059-y>.

[55] Guerrero F, Lucar N, Garvich Claux M, Chiappe M, Perez-Lu J, Hindin MJ, et al. Developing an SMS text message intervention on sexual and reproductive health with adolescents and youth in Peru. *Reprod Health*. 2020;17:116. <https://doi.org/10.1186/s12978-020-00943-6>.

[56] Gonsalves L, Hindin MJ, Bayer A, Carcamo CP, Gichangi P, Habib N, et al. Protocol of an open, three-arm, individually randomized trial assessing the effect of delivering sexual and reproductive health information to young people (aged 13-24) in Kenya and Peru via mobile phones: adolescent/youth reproductive mobile access and delivery initiative for love and life outcomes (ARMADILLO) study stage 2. *Reprod Health.* 2018;15:126. <https://doi.org/10.1186/s12978-018-0568-6>.

[57] Perez-Lu JE, Guerrero F, Cárcamo CP, Alburqueque M, Chiappe M, Hindin MJ, et al. The ARMADILLO text message intervention to improve the sexual and reproductive health knowledge of adolescents in Peru: Results of a randomized controlled trial. *PloS One.* 2022;17:e0262986. <https://doi.org/10.1371/journal.pone.0262986>.

[58] Gichangi P, Gonsalves L, Mwaisaka J, Thiongo M, Habib N, Waithaka M, et al. Busting contraception myths and misconceptions among youth in Kwale County, Kenya: results of a digital health randomised control trial. *BMJ Open.* 2022;12:e047426. <https://doi.org/10.1136/bmjopen-2020-047426>.

[59] Gonsalves L, Njeri WW, Schroeder M, Mwaisaka J, Gichangi P. Research and Implementation Lessons Learned From a Youth-Targeted Digital Health Randomized Controlled Trial (the ARMADILLO Study). *JMIR MHealth UHealth.* 2019;7:e13005. <https://doi.org/10.2196/13005>.

[60] Mwaisaka J, Gonsalves L, Thiongo M, Waithaka M, Sidha H, Alfred O, et al. Young People’s Experiences Using an On-Demand Mobile Health Sexual and Reproductive Health Text Message Intervention in Kenya: Qualitative Study. *JMIR MHealth UHealth.* 2021;9:e19109. <https://doi.org/10.2196/19109>.

- ***CAN-DO, Australia***

[61] Rouf A, Allman-Farinelli M. Messaging for Interventions Aiming to Improve Calcium Intake in Young Adults-A Mixed Methods Study. *Nutrients.* 2018;10:1673. <https://doi.org/10.3390/nu10111673>.

[62] Rouf A, Nour M, Allman-Farinelli M. Improving Calcium Knowledge and Intake in Young Adults Via Social Media and Text Messages: Randomized Controlled Trial. *JMIR MHealth UHealth.* 2020;8:e16499. <https://doi.org/10.2196/16499>.

- ***Contraception Choices, United Kingdom***

[63] Stephenson J, Bailey JV, Blandford A, Brima N, Copas A, D’Souza P, et al. An interactive website to aid young women’s choice of contraception: feasibility and efficacy RCT. *Health Technol Assess*. 2020;24:1–44. <https://doi.org/10.3310/hta24560>.

[64] Stephenson J, Bailey JV, Gubijev A, D’Souza P, Oliver S, Blandford A, et al. An interactive website for informed contraception choice: randomised evaluation of Contraception Choices. *Digit Health*. 2020;6:2055207620936435. <https://doi.org/10.1177/2055207620936435>.

[65] Bailey JV, Bennett KF, Gubijev A, Shawe J, Stephenson J. Participant views and experiences of sexual health research: The Contraception Choices online trial. *Digit Health.* 2021;7:20552076211033424. <https://doi.org/10.1177/20552076211033424>.

- ***CyberRwanda, Rwanda***

[66] Nolan C, Packel L, Hope R, Levine J, Baringer L, Gatare E, et al. Design and impact evaluation of a digital reproductive health program in Rwanda using a cluster randomized design: study protocol. *BMC Public Health.* 2020;20:1701. <https://doi.org/10.1186/s12889-020-09746-7>.

[67] Ippoliti N, Sekamana M, Baringer L, Hope R. Using Human-Centered Design to Develop, Launch, and Evaluate a National Digital Health Platform to Improve Reproductive Health for Rwandan Youth. *Glob Health Sci Pract.* 2021;9:S244–60. <https://doi.org/10.9745/GHSP-D-21-00220>.

[68] Hémono R, Packel L, Gatare E, Baringer L, Ippoliti N, McCoy SI, et al. Digital self-care for improved access to family planning and reproductive health services among adolescents in Rwanda: preliminary findings from a pilot study of CyberRwanda. *Sex Reprod Health Matters.* 2022;29:2110671. <https://doi.org/10.1080/26410397.2022.2110671>.

[69] Hémono R, Gatare E, Kayitesi L, Packel L, Hunter LA, Kunesh J, et al. CyberRwanda’s Pathway to Impact: Results From a Cluster-Randomized Trial of Adolescent Family Planning Knowledge, Beliefs, Self-Efficacy, and Behavior. *J Adolesc Health*. 2024;74:1239–48. <https://doi.org/10.1016/j.jadohealth.2024.01.035>.

- ***eCHECKUP TO GO: High School, United States of America***

[70] Doumas DM. Web-based personalized feedback: is this an appropriate approach for reducing drinking among high school students? *J Subst Abuse Treat.* 2015;50:76–80. <https://doi.org/10.1016/j.jsat.2014.09.005>.

[71] Doumas DM, Esp S, Johnson J, Trull R, Shearer K. The eCHECKUP TO GO for High School: Impact on risk factors and protective behavioral strategies for alcohol use. *Addict Behav.* 2017;64:93–100. <https://doi.org/10.1016/j.addbeh.2016.08.030>.

[72] Doumas DM, Esp S, Flay B, Bond L. A Randomized Controlled Trial Testing the Efficacy of a Brief Online Alcohol Intervention for High School Seniors. *J Stud Alcohol Drugs*. 2017;78:706–15. <https://doi.org/10.15288/jsad.2017.78.706>.

[73] Doumas DM, Esp S. Reducing Alcohol-Related Consequences Among High School Seniors: Efficacy of a Brief, Web-Based Intervention. *J Couns Dev*. 2019;97:53–61. <https://doi.org/10.1002/jcad.12235>.

[74] Doumas DM, Esp S, Turrisi R, Bond L. A Randomized Controlled Trial of the eCHECKUP to GO for High School Seniors across the Academic Year. *Subst Use Misuse.* 2021;56:1923–32. <https://doi.org/10.1080/10826084.2021.1958862>.

[75] Doumas DM, Esp S, Turrisi R, Bond L, Porchia S, Flay B. Sex differences in the acceptability and short-term outcomes of a web-based personalized feedback alcohol intervention for high school seniors. *Psychol Sch.* 2020;57:1724–40. <https://doi.org/10.1002/pits.22422>.

[76] Doumas DM, Esp S, Turrisi R, Bond L, Flay B. Efficacy of the eCHECKUP TO GO for High School Seniors: Sex Differences in Risk Factors, Protective Behavioral Strategies, and Alcohol Use. *J Stud Alcohol Drugs*. 2020;81:135–43. <https://doi.org/10.15288/jsad.2020.81.135>.

- ***Fume, Finland and Portugal***

[77] Parisod H, Pakarinen A, Axelin A, Danielsson-Ojala R, Smed J, Salanterä S. Designing a Health-Game Intervention Supporting Health Literacy and a Tobacco-Free Life in Early Adolescence. *Games Health J.* 2017;6:187–99. <https://doi.org/10.1089/g4h.2016.0107>.

[78] Parisod H, Pakarinen A, Axelin A, Löyttyniemi E, Smed J, Salanterä S. Feasibility of mobile health game “Fume” in supporting tobacco-related health literacy among early adolescents: A three-armed cluster randomized design. *Int J Med Inf.* 2018;113:26–37. <https://doi.org/10.1016/j.ijmedinf.2018.02.013>.

[79] Pinto DL, Parisod H, Nyman J, Barroso TMMD de A. Effectiveness of the Portuguese version of Fume in adolescents’ health literacy about tobacco. *Rev Lat Am Enfermagem*. 2022;30:e3513. <https://doi.org/10.1590/1518-8345.5455.3513>.

- ***Girl2Girl, United States of America***

[80] Ybarra ML, Price-Feeney M, Prescott T, Goodenow C, Saewyc E, Rosario M. Girl2Girl: How to develop a salient pregnancy prevention program for cisgender sexual minority adolescent girls. *J Adolesc*. 2020;85:41–58. <https://doi.org/10.1016/j.adolescence.2020.09.006>.

[81] Ybarra M, Goodenow C, Rosario M, Saewyc E, Prescott T. An mHealth Intervention for Pregnancy Prevention for LGB Teens: An RCT. *Pediatrics.* 2021;147:e2020013607. <https://doi.org/10.1542/peds.2020-013607>.

[82] Ybarra M, Rosario M, Saewyc E, Goodenow C, Dunsiger S. One-Year Follow-up After a Pregnancy Prevention Intervention for LGB1 Teens: An RCT. *Pediatrics*. 2023;151:e2022059172. <https://doi.org/10.1542/peds.2022-059172>.

[83] Ybarra ML, Saewyc E, Rosario M, Dunsiger S. Subgroup Analyses of Girl2Girl, a Text Messaging-Based Teen Pregnancy Prevention Program for Sexual Minority Girls: Results from a National RCT. *Prev Sci.* 2023;24:292–9. <https://doi.org/10.1007/s11121-023-01493-6>.

- ***Global Consent, Vietnam***

[84] Yount KM, Minh TH, Trang QT, Cheong YF, Bergenfeld I, Sales JM. Preventing sexual violence in college men: a randomized-controlled trial of GlobalConsent. *BMC Public Health.* 2020;20:1331. <https://doi.org/10.1186/s12889-020-09454-2>.

[85] Yount KM, Cheong YF, Bergenfeld I, Trang QT, Sales JM, Li Y, et al. Impacts of GlobalConsent, a Web-Based Social Norms Edutainment Program, on Sexually Violent Behavior and Bystander Behavior Among University Men in Vietnam: Randomized Controlled Trial. *JMIR Public Health Surveill*. 2023;9:e35116. <https://doi.org/10.2196/35116>.

[86] Yount KM, Bergenfeld I, Anderson KM, Trang QT, Sales JM, Cheong YF, et al. Theoretical mediators of GlobalConsent: An adapted web-based sexual violence prevention program for university men in Vietnam. *Soc Sci Med;* 2022;313:115402. <https://doi.org/10.1016/j.socscimed.2022.115402>.

[87] Yount KM, Anderson KM, Trang QT, Bergenfeld I. Preventing sexual violence in Vietnam: qualitative findings from high school, university, and civil society key informants across regions. *BMC Public Health.* 2023;23:1114. <https://doi.org/10.1186/s12889-023-15973-5>.

- ***HEART for Teens, United States of America***

[88] Javidi H, Widman L, Lipsey N, Brasileiro J, Javidi F, Jhala A. Redeveloping a Digital Sexual Health Intervention for Adolescents to Allow for Broader Dissemination: Implications for HIV and STD Prevention. *AIDS Educ Prev*. 2021;33:89–102. <https://doi.org/10.1521/aeap.2021.33.2.89>.

[89] McCrimmon J, Widman L, Javidi H, Brasileiro J, Hurst J. Evaluation of a Brief Online Sexual Health Program for Adolescents: A Randomized Controlled Trial. *Health Promot Pract.* 2023:15248399231162379. <https://doi.org/10.1177/15248399231162379>.

- ***HOPE, Singapore***

[90] Tay JL, Goh Y-SS, Klainin-Yobas P. Online HOPE intervention on mental health literacy among youths in Singapore: An RCT protocol. *J Adv Nurs*. 2020;76:1831–9. <https://doi.org/10.1111/jan.14393>.

[91] Tay JL, Goh YSS, Sim K, Klainin-Yobas P. Impact of the HOPE Intervention on Mental Health Literacy, Psychological Well-Being and Stress Levels amongst University Undergraduates: A Randomised Controlled Trial. *Int J Environ Res Public Health.* 2022;19:9772. <https://doi.org/10.3390/ijerph19159772>.

[92] Tay JL. Online HOPE intervention on help-seeking attitudes and intentions among young adults in Singapore: A randomized controlled trial and process evaluation. *Arch Psychiatr Nurs.* 2022;41:286–94. <https://doi.org/10.1016/j.apnu.2022.09.008>.

- ***Intellicare for College Students, United States of America***

[93] Cohen KA, Graham AK, Lattie EG. Aligning students and counseling centers on student mental health needs and treatment resources. *J Am Coll Health.* 2022;70:724–32. <https://doi.org/10.1080/07448481.2020.1762611>.

[94] Lattie E, Cohen KA, Winquist N, Mohr DC. Examining an App-Based Mental Health Self-Care Program, IntelliCare for College Students: Single-Arm Pilot Study. *JMIR Ment Health*. 2020;7:e21075. <https://doi.org/10.2196/21075>.

[95] Lattie EG, Cohen KA, Hersch E, Williams KDA, Kruzan KP, MacIver C, et al. Uptake and effectiveness of a self-guided mobile app platform for college student mental health*. Internet Interv.* 2022;27:100493. <https://doi.org/10.1016/j.invent.2021.100493>.

- ***Media Aware (High School), United States of America***

[96] Scull T, Malik C, Morrison A, Keefe E. Promoting Sexual Health in High School: A Feasibility Study of A Web-based Media Literacy Education Program. *J Health Commun.* 2021;26:147–60. <https://doi.org/10.1080/10810730.2021.1893868>.

[97] Scull TM, Malik CV, Morrison A, Keefe EM. Study protocol for a randomized controlled trial to evaluate a web-based comprehensive sexual health and media literacy education program for high school students. *Trials*. 2020;21:50. <https://doi.org/10.1186/s13063-019-3992-1>.

[98] Scull TM, Dodson CV, Geller JG, Reeder LC, Stump KN. A Media Literacy Education Approach to High School Sexual Health Education: Immediate Effects of Media Aware on Adolescents’ Media, Sexual Health, and Communication Outcomes. *J Youth Adolesc.* 2022;51:708–23. <https://doi.org/10.1007/s10964-021-01567-0>.

- ***mi.spot, Australia***

[99] Matar JL, Maybery DJ, McLean LA, Reupert A. Web-Based Health Intervention for Young People Who Have a Parent with a Mental Illness: Delphi Study Among Potential Future Users. *J Med Internet Res*. 2018;20:e10158. <https://doi.org/10.2196/10158>.

[100] Reupert A, Bartholomew C, Cuff R, Foster K, Matar J, Maybery DJ, et al. An Online Intervention to Promote Mental Health and Wellbeing for Young Adults Whose Parents Have Mental Illness and/or Substance Use Problems: Theoretical Basis and Intervention Description. *Front Psychiatry.* 2019;10:59. <https://doi.org/10.3389/fpsyt.2019.00059>.

[32] Reupert A, Maybery D, Bartholomew C, Cuff R, Foster K, Matar J, et al. The Acceptability and Effectiveness of an Online Intervention for Youth With Parents With a Mental Illness and/or Substance Use Issue. *J Adolesc Health.* 2020;66:551–8. <https://doi.org/10.1016/j.jadohealth.2019.11.309>.

[101] Maybery D, Reupert A, Bartholomew C, Cuff R, Duncan Z, Foster K, et al. A Web-Based Intervention for Young Adults Whose Parents Have a Mental Illness or Substance Use Concern: Protocol for a Randomized Controlled Trial. *JMIR Res Protoc.* 2020;9:e15626. <https://doi.org/10.2196/15626>.

[102] Maybery D, Reupert A, Bartholomew C, Cuff R, Duncan Z, McAuliffe C, et al. An online intervention for 18-25-year-old youth whose parents have a mental illness and/or substance use disorder: A pilot randomized controlled trial. *Early Interv Psychiatry.* 2022;16:1249–58. <https://doi.org/10.1111/eip.13274>.

- ***MyPEEPS, United States of America***

[103] Schnall R, Kuhns LM, Hidalgo MA, Powell D, Thai J, Hirshfield S, et al. Adaptation of a Group-Based HIV RISK Reduction Intervention to a Mobile App for Young Sexual Minority Men. *AIDS Educ Prev*. 2018;30:449–62. <https://doi.org/10.1521/aeap.2018.30.6.449>.

[104] Cho H, Powell D, Pichon A, Thai J, Bruce J, Kuhns LM, et al. A Mobile Health Intervention for HIV Prevention Among Racially and Ethnically Diverse Young Men: Usability Evaluation. *JMIR MHealth UHealth.* 2018;6:e11450. <https://doi.org/10.2196/11450>.

[105] Ignacio M, Garofalo R, Pearson C, Kuhns LM, Bruce J, Scott Batey D, et al. Pilot feasibility trial of the MyPEEPS mobile app to reduce sexual risk among young men in 4 cities. *JAMIA Open.* 2019;2:272–9. <https://doi.org/10.1093/jamiaopen/ooz008>.

[106] Kuhns LM, Garofalo R, Hidalgo M, Hirshfield S, Pearson C, Bruce J, et al. A randomized controlled efficacy trial of an mHealth HIV prevention intervention for sexual minority young men: MyPEEPS mobile study protocol. *BMC Public Health.* 2020;20:65. <https://doi.org/10.1186/s12889-020-8180-4>.

[107] Schnall R, Kuhns LM, Pearson C, Batey DS, Bruce J, Hidalgo MA, et al. Efficacy of MyPEEPS Mobile, an HIV Prevention Intervention Using Mobile Technology, on Reducing Sexual Risk Among Same-Sex Attracted Adolescent Males: A Randomized Clinical Trial. *JAMA Netw Open*. 2022;5:e2231853. <https://doi.org/10.1001/jamanetworkopen.2022.31853>.

[108] Cordoba E, Idnay B, Garofalo R, Kuhns LM, Pearson C, Bruce J, et al. Examining the Information Systems Success (ISS) of a mobile sexual health app (MyPEEPS Mobile) from the perspective of very young men who have sex with men (YMSM). *Int J Med Inf.* 2021;153:104529. <https://doi.org/10.1016/j.ijmedinf.2021.104529>.

[109] Anderson A, Karczmar A, Kuhns LM, Garofalo R, Radix A, Bruce J, et al. A Qualitative Study to Inform Adaptation of MyPEEPS Mobile for Transmasculine Youth. *J Health Care Poor Underserved.* 2022;33:301–16. <https://doi.org/10.1353/hpu.2022.0022>.

- ***Outsmart HPV, United States of America***

[110] Reiter PL, Katz ML, Bauermeister JA, Shoben AB, Paskett ED, McRee A-L. Increasing Human Papillomavirus Vaccination Among Young Gay and Bisexual Men: A Randomized Pilot Trial of the Outsmart HPV Intervention. *LGBT Health*. 2018;5:325–9. <https://doi.org/10.1089/lgbt.2018.0059>.

[111] McRee A-L, Shoben A, Bauermeister JA, Katz ML, Paskett ED, Reiter PL. Outsmart HPV: Acceptability and short-term effects of a web-based HPV vaccination intervention for young adult gay and bisexual men. *Vaccine.* 2018;36:8158–64. <https://doi.org/10.1016/j.vaccine.2018.01.009>.

[112] Reiter PL, Gower AL, Kiss DE, Malone MA, Katz ML, Bauermeister JA, et al. A Web-Based Human Papillomavirus Vaccination Intervention for Young Gay, Bisexual, and Other Men Who Have Sex With Men: Protocol for a Randomized Controlled Trial. *JMIR Res Protoc*. 2020;9:e16294. <https://doi.org/10.2196/16294>.

[113] Reiter PL, Gower AL, Kiss DE, Shoben AB, Katz ML, Bauermeister JA, et al. Effects of a web-based HPV vaccination intervention on cognitive outcomes among young gay, bisexual, and other men who have sex with men. *Hum Vaccines Immunother.* 2022;18:2114261. <https://doi.org/10.1080/21645515.2022.2114261>.

[114] Reiter PL, Gower AL, Kiss DE, Shoben AB, Katz ML, Bauermeister JA, et al. Efficacy of the Outsmart HPV Intervention: A Randomized Controlled Trial to Increase HPV Vaccination among Young Gay, Bisexual, and Other Men Who Have Sex with Men. *Cancer Epidemiol Biomark Prev*. 2023;32:760–7. <https://doi.org/10.1158/1055-9965.EPI-23-0007>.

- ***PACT (Promoting Affirmative Consent among Teens), United States of America***

[115] Javidi H, Widman L, Maheux AJ, McCrimmon J, Evans-Paulson R, Becker W. PACT: Developing and Evaluating a Digital Sexual Consent Program for Youth. *J Sex Res*. 2024;61:466–80. <https://doi.org/10.1080/00224499.2023.2208560>.

- ***PlayTest!, United States of America***

[116] Pendergrass T, Hieftje K, Duncan L, Fiellin L. Videogame intervention to encourage HIV testing and counseling among adolescents. *mHealth.* 2020;6. <https://doi.org/10.21037/mhealth.2020.01.05>.

[117] Boomer TP, Larkin K, Duncan LR, Fernandes C-SF, Fiellin LE. A Serious Video Game Targeting HIV Testing and Counseling: A Randomized Controlled Trial. *J Adolesc Health.* 2024;74:252–9. <https://doi.org/10.1016/j.jadohealth.2023.08.016>.

- ***Pop4Teens, United States of America***

[118] Moore SK, Grabinski M, Bessen S, Borodovsky JT, Marsch LA. Web-Based Prescription Opioid Abuse Prevention for Adolescents: Program Development and Formative Evaluation. *JMIR Form Res*. 2019;3:e12389. <https://doi.org/10.2196/12389>.

[119] Marsch LA, Moore SK, Grabinski M, Bessen SY, Borodovsky J, Scherer E. Evaluating the Effectiveness of a Web-Based Program (POP4Teens) to Prevent Prescription Opioid Misuse Among Adolescents: Randomized Controlled Trial. *JMIR Public Health Surveill.* 2021;7:e18487. <https://doi.org/10.2196/18487>.

- ***REAL Media, United States of America***

[120] Ray AE, Greene K, Hecht ML, Barriage SC, Miller-Day M, Glenn SD, et al. An E-Learning Adaptation of an Evidence-Based Media Literacy Curriculum to Prevent Youth Substance Use in Community Groups: Development and Feasibility of REAL Media. *JMIR Form Res*. 2019;3:e12132. <https://doi.org/10.2196/12132>.

[121] Ray AE, Greene K, Pristavec T, Hecht ML, Miller-Day MA, Banerjee SC. Exploring Indicators of Engagement in Online Learning as Applied to Adolescent Health Prevention: A Pilot Study of REAL media. *Educ Technol Res Dev.* 2020;68:3143–63. <https://doi.org/10.1007/s11423-020-09813-1>.

[122] Greene K, Ray AE, Choi HJ, Glenn SD, Lyons RE, Hecht ML. Short term effects of the REAL media e-learning media literacy substance prevention curriculum: An RCT of adolescents disseminated through a community organization. *Drug Alcohol Depend.* 2020;214:108170. <https://doi.org/10.1016/j.drugalcdep.2020.108170>.

[123] Peña-Alves S, Greene K, Ray AE, Glenn SD, Hecht ML, Banerjee SC. “Choose Today, Live Tomorrow”: A Content Analysis of Anti-Substance Use Messages Produced by Adolescents. *J Health Commun.* 2019;24:592–602. <https://doi.org/10.1080/10810730.2019.1639858>.

[124] Greene K, Choi HJ, Glenn SD, Ray AE, Hecht ML. The Role of Engagement in Effective, Digital Prevention Interventions: the Function of Engagement in the REAL Media Substance Use Prevention Curriculum. *Prev Sci Off J Soc Prev Res*. 2021;22:247–58. <https://doi.org/10.1007/s11121-020-01181-9>.

- ***SMART, United States of America***

[125] Li DH, Moskowitz DA, Macapagal K, Saber R, Mustanski B. Using Intervention Mapping to Developmentally Adapt an Online HIV Risk Reduction Program for Adolescent Men Who Have Sex with Men. *Prev Sci Off J Soc Prev Res*. 2020;21:885–97. <https://doi.org/10.1007/s11121-020-01148-w>.

[126] Ventuneac A, Li DH, Mongrella MC, Moskowitz DA, Weingardt KR, Brown CH, et al. Exploring potential implementation barriers and facilitators of the SMART Program, a stepped-care package of eHealth HIV prevention interventions for adolescent men who have sex with men. *Sex Res Soc Policy.* 2020;17:378–88. <https://doi.org/10.1007/s13178-019-00402-3>.

[127] Mustanski B, Moskowitz DA, Moran KO, Newcomb ME, Macapagal K, Rodriguez-Díaz C, et al. Evaluation of a Stepped-Care eHealth HIV Prevention Program for Diverse Adolescent Men Who Have Sex With Men: Protocol for a Hybrid Type 1 Effectiveness Implementation Trial of SMART. *JMIR Res Protoc.* 2020;9:e19701. <https://doi.org/10.2196/19701>.

[128] Saber R, Mongrella M, Matson M, Carty-Fickes E, Novotny J, Pirog S, et al. Use of a custom testing center locator tool to improve STI and HIV testing rates in adolescent men who have sex with men as part of an online sexual health program. *Procedia Comput Sci.* 2022;206:92–100. <https://doi.org/10.1016/j.procs.2022.09.088>.

[129] Mustanski B, Saber R, Macapagal K, Matson M, Laber E, Rodriguez-Diaz C, et al. Effectiveness of the SMART Sex Ed program among 13-18 year old English and Spanish speaking adolescent men who have sex with men. *AIDS Behav*. 2023;27:733–44. <https://doi.org/10.1007/s10461-022-03806-2>.

[130] Sinno J, Macapagal K, Mustanski B. Social Media and Online Dating Safety Practices by Adolescent Sexual and Gender Diverse Men: Mixed-Methods Findings From the SMART Study. *J Adolesc Health.* 2024;74:113–22. <https://doi.org/10.1016/j.jadohealth.2023.07.030>.

- ***Topity, Brazil***

[131] Matheson EL, Smith HG, Amaral ACS, Meireles JFF, Almeida MC, Mora G, et al. Improving body image at scale among Brazilian adolescents: study protocol for the co-creation and randomised trial evaluation of a chatbot intervention. *BMC Public Health.* 2021;21:2135. <https://doi.org/10.1186/s12889-021-12129-1>.

[132] Matheson EL, Smith HG, Amaral ACS, Meireles JFF, Almeida MC, Linardon J, et al. Using Chatbot Technology to Improve Brazilian Adolescents’ Body Image and Mental Health at Scale: Randomized Controlled Trial. *JMIR MHealth UHealth.* 2023;11:e39934. <https://doi.org/10.2196/39934>.

- ***Warna-Warni Waktu, Indonesia***

[133] Garbett KM, Craddock N, Haywood S, Nasution K, White P, Saraswati LA, et al. A Novel, Scalable Social Media–Based Intervention (“Warna-Warni Waktu”) to Reduce Body Dissatisfaction Among Young Indonesian Women: Protocol for a Parallel Randomized Controlled Trial. *JMIR Res Protoc*. 2022;11:e33596. <https://doi.org/10.2196/33596>.

[134] Garbett KM, Haywood S, Craddock N, Gentili C, Nasution K, Saraswati LA, et al. Evaluating the Efficacy of a Social Media–Based Intervention (Warna-Warni Waktu) to Improve Body Image Among Young Indonesian Women: Parallel Randomized Controlled Trial. *J Med Internet Res.* 2023;25:e42499. <https://doi.org/10.2196/42499>.
