# Supplementary material for: A scoping review of outcome selection and accuracy of conclusions in complex digital health interventions for young people (2017–2023): methodological proposals for population health intervention research
Source: BMC Med. 2025 Jul 2;23:400. doi: 10.1186/s12916-025-04245-1 (PMC12224660; doi:10.1186/s12916-025-04245-1)
Supplement: Supplementary file 6 — Additional file 6: Table S4. Outcomes’ characteristics. Table S5. Outcomes’ measurement methods. [file 12916_2025_4245_MOESM6_ESM.docx]

## Additional File 6. Characteristics of retrieved outcomes (n=293)

**Table S4. Characteristics of all retrieved outcomes across the 26 interventions (n=293)**

|  | **Effectiveness outcomes** | **Process outcomes** | **Economic outcomes** | **Total** |
| --- | --- | --- | --- | --- |
| **All retrieved outcomes**, n (%)**^†^** | **171 (58)** | **107 (37)** | **15 (5)** | **293** |
| Primary outcomes | 36 (97) | 1 (3) | - | 37 |
| Secondary outcomes | 48 (91) | 5 (9) | - | 53 |
| Non-hierarchised outcomes | 87 (43) | 101 (50) | 15 (7) | 203 |
| **Reported outcomes only**, n (%)**^‡^** | **164 (65)** | **78 (31)** | **9 (4)** | **251^x^** |
| Primary outcomes | 33 (97) | 1 (3) | - | 34 |
| Secondary outcomes | 45 (90) | 5 (10) | - | 50 |
| Non-hierarchised outcomes | 86 (51) | 72 (43) | 9 (5) | 167 |

† The outcome count includes all retrieved outcomes, including those found solely in protocols. Row percentages. Percentages may not add up to 100 due to rounding.

‡ The outcome count includes only reported outcomes, excluding those found solely in protocols. Row percentages. Percentages may not add up to 100 due to rounding.

^x^ This represents the number of outcomes reported in Figure 2 of the main manuscript, excluding 42 outcomes that were declared in protocols but not reported. These 42 outcomes are shown in grey in Figure 2.

**Table S5. Measurement methods for reported outcomes (n=251)**

|  | **Effectiveness outcomes** | **Process outcomes** | **Economic outcomes** | **Total** |
| --- | --- | --- | --- | --- |
| **Quantitative outcomes**, n (%)* | **164 (100)** | **50 (100)** | **9 (100)** | **223** |
| Measurement instrument |  |  |  |  |
| Scale | 92 (56) | 25 (50) | - | 117 |
| *Subjective measure†* | *86 (93)* | *25 (100)* | *-* | *111* |
| *Validated‡* | *47 (55)* | *2 (8)* | *-* | *49* |
| Questionnaire | 66 (40) | 6 (12) | - | 72 |
| *Subjective measure†* | *30 (45)* | *4 (67)* | *-* | *34* |
| *Validated‡* | *17 (57)* | *1 (25)* | *-* | *18* |
| Digital device used for the intervention | 0 (0) | 19 (38) | - | 19 |
| Questionnaire + Digital device | 3 (2) | 0 (0) | - | 3 |
| Medical device | 1 (0) | 0 (0) | - | 1 |
| Health service data logs | 1 (0) | 0 (0) | - | 1 |
| Vignettes or pictorial tools | 1 (0) | 0 (0) | - | 1 |
| Cost estimation tools | - | - | 9 (100) | 9 |
| Data collection personnel |  |  |  |  |
| Self-reported | 153 (93) | 27 (54) | - | 180 |
| Researcher-reported | 7 (4) | 4 (8) | 9 (100) | 20 |
| Reported by parents or guardians | 1 (0) | 0 (0) | - | 1 |
| Self-reported, complemented by a  medical/digital device | 3 (2) | 0 (0) | - | 3 |
| Digital device used for the intervention | 0 (0) | 19 (38) | - | 19 |
| **Qualitative outcomes**, n (%)* | **0** (0) | **28** (56) | **0** (0) | **28** |
| Measurement instrument |  |  |  |  |
| Interviews | - | 19 (38) | - | 19 |
| Open-ended questionnaire field | - | 9 (18) | - | 9 |
| Data collection personnel |  |  |  |  |
| Self-reported | - | 28 (56) | - | 28 |

-: not applicable.

*Column percentages. Percentages may not add up to 100 due to rounding.

† Among outcomes measured with a scale or questionnaire, the count of subjective endpoints.

‡ Among subjective outcomes, the count of endpoints assessed with a validated measure.
