# Supplementary material for: A scoping review of outcome selection and accuracy of conclusions in complex digital health interventions for young people (2017–2023): methodological proposals for population health intervention research
Source: BMC Med. 2025 Jul 2;23:400. doi: 10.1186/s12916-025-04245-1 (PMC12224660; doi:10.1186/s12916-025-04245-1)
Supplement: Supplementary file 7 — Additional file 7: Figure S1. Role of outcomes in conclusions. [file 12916_2025_4245_MOESM7_ESM.docx]

## Additional File 7. Role of outcomes in conclusions on intervention success

**
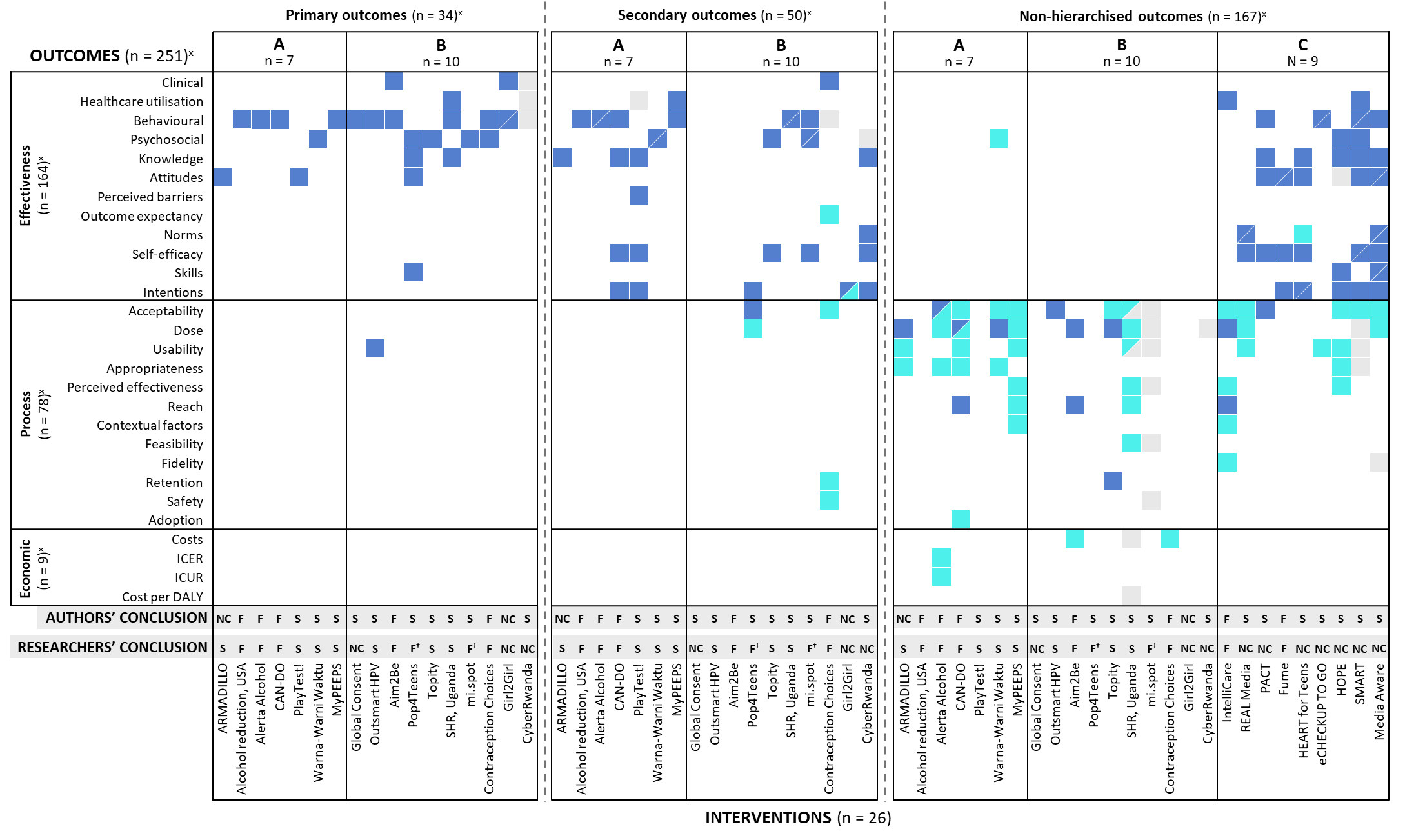
**

^x^ Number of reported outcomes, excludes the 42 outcomes mentioned in protocols only. Intervention names are listed at the bottom of the figure. **Intervention categorisation:** A: intervention studies defining a unique primary outcome, with or without additional outcomes. B: intervention studies defining multiple primary outcomes, with or without additional outcomes. C: intervention studies defining multiple non-hierarchised outcomes. Interventions in categories A and B are displayed across the three outcome hierarchical positions – primary, secondary, and non-hierarchised – and therefore appear three times in the figure. **Outcome role in conclusions:** Dark blue: outcomes used by the authors to determine intervention success (i.e. reported in the justification of intervention success or failure). Light blue: outcomes measured but not further used to determine intervention success (i.e. reported in the *Results* section of articles only). Grey: outcomes in protocols only (n=42), not included in the total outcome count. **Conclusions on intervention success**: S: success. F: failure. NC: non-conclusive. F†: spin (when authors reported success despite all primary outcomes being not statistically significant). **Abbreviations:** ICER: incremental cost-effectiveness ratio; ICUR: incremental cost-utility ratio; DALY: disability-adjusted life years; SRH: Sexual and Reproductive Health. **How to read the figure:** for example, in the ARMADILLO study (category A intervention), the two effectiveness outcomes (knowledge and attitudes) and one process outcome (dose) were used in the conclusion regarding intervention success (dark blue), whereas the two remaining process outcomes (usability, and appropriateness) were only presented in the *Results* section and not used to conclude on intervention success (light blue).

**Figure S1. Role of outcomes in conclusions on digital health interventions’ success (n=26)**
