# Supplementary material for: A scoping review of outcome selection and accuracy of conclusions in complex digital health interventions for young people (2017–2023): methodological proposals for population health intervention research
Source: BMC Med. 2025 Jul 2;23:400. doi: 10.1186/s12916-025-04245-1 (PMC12224660; doi:10.1186/s12916-025-04245-1)
Supplement: Supplementary file 8 — Additional file 8: Table S6. Inter-rater agreement on conclusions. Table S7. Inter-rater agreement on conclusions by intervention categorisation. [file 12916_2025_4245_MOESM8_ESM.docx]

## Additional File 8. Agreement percentages on intervention conclusions

**Table S6. Inter-rater agreement on intervention conclusions (n=26)**

|  |  | **Authors’ conclusions, n (%)*** | | |
| --- | --- | --- | --- | --- |
|  |  | Success | Failure | Non-conclusive |
| **Researchers’ conclusions,**  **n (%)*** | Success | 6 (23) | - | **1 (4)** |
|  | Failure | **2 (8)** | 7 (27) | - |
|  | Non-conclusive | **9 (35)** | - | 1 (4) |

*Percentages do not add up to 100 due to rounding.

Bolded results indicate situations where the authors and the research team reached different conclusions about intervention success.

Explanation of results. In 54% of interventions, researchers agreed with authors’ conclusions regarding intervention success. In 47% of interventions, researchers disagreed with authors’ conclusions.

Two interventions (8%) were considered successful in achieving their objectives by the authors but were deemed unsuccessful (i.e. failures) in achieving objectives by the researchers. These two interventions were classified as spin by the researchers.

**Table S7. Inter-rater agreement on intervention conclusions (n=26), stratified by intervention categorisation**

| **Intervention studies using a unique primary outcome (n=7)** | | | | |
| --- | --- | --- | --- | --- |
|  |  | **Authors’ conclusions, n** | | |
|  |  | Success | Failure | Non-conclusive |
| **Researchers’ conclusions, n** | Success | 3 | - | **1** |
|  | Failure | - | 3 | - |
|  | Non-conclusive | - | - | - |
| **Intervention studies using multiple primary outcomes (n=10)** | | | | |
|  |  | **Authors’ conclusions, n** | | |
|  |  | Success | Failure | Non-conclusive |
| **Researchers’ conclusions, n** | Success | 3 | - | - |
|  | Failure | **2** | 2 | - |
|  | Non-conclusive | **2** | - | 1 |
| **Intervention studies using multiple non-hierarchised outcomes (n=9)** | | | | |
|  |  | **Authors’ conclusions, n** | | |
|  |  | Success | Failure | Non-conclusive |
| **Researchers’ conclusions, n** | Success | - | - | - |
|  | Failure | - | 2 | - |
|  | Non-conclusive | **7** | - | - |

Bolded results indicate situations where the authors and the research team reached different conclusions about intervention success.
