# Supplementary material for: A scoping review of outcome selection and accuracy of conclusions in complex digital health interventions for young people (2017–2023): methodological proposals for population health intervention research
Source: BMC Med. 2025 Jul 2;23:400. doi: 10.1186/s12916-025-04245-1 (PMC12224660; doi:10.1186/s12916-025-04245-1)
Supplement: Supplementary file 9 — Additional file 9: Table S8. Outcomes assessment in the sub-analysis. [file 12916_2025_4245_MOESM9_ESM.docx]

## Additional File 9. Description of outcomes collected to assess the impact of outcome hierarchical position on intervention conclusions

**Table S8. Outcomes assessed in the evaluation of three sexual and reproductive health interventions to illustrate the impact of outcome hierarchical position on intervention conclusions**

|  | **Intervention 1** | | | **Intervention 2** | | | **Intervention 3** | | |
| --- | --- | --- | --- | --- | --- | --- | --- | --- | --- |
| **General characteristics** | | | | | | | | | |
| Objective | HIV prevention | | | Preventing unwanted pregnancy | | | HIV prevention | | |
| Intervention theories | Social-Personal framework, Social Learning Theory | | | IMB skills model for HIV prevention | | | IMB skills model for HIV prevention | | |
| Target population | Men who have sex with men | | | Cis-gender, lesbian and bisexual girls | | | Men who have sex with men | | |
| Target age | 13-18 | | | 14-18 | | | 13-18 | | |
| Technology | App | | | Text messages | | | App + Live sessions | | |
| Intervention duration | 3 months | | | 4,5 months | | | 9 months | | |
| Implementation setting | Daily life | | | Daily life | | | Daily life | | |
| **Methods** | | | | | | | | | |
| Study design | 2-arm parallel RCT | | | 2-arm parallel RCT | | | Pretest-posttest design | | |
| Measurement time points | M3, M6, M9 | | | M3, M6, M9, M12 | | | M3 | | |
| N participants: intervention/control (M3) | 380 / 381 | | | 389 / 410 | | | 983 / 983 | | |
| **Outcomes** | | | | | | | | | |
| Original outcome hierarchisation | Unique primary outcome | | | Multiple primary outcomes | | | Non-hierarchised outcomes | | |
| N outcomes assessed | 16 | | | 7 | | | 18 | | |
| N outcomes statistically tested | 8 | | | 7 | | | 18 | | |
| N primary outcomes | 1 | | | 4 | | | 0 | | |
| N secondary outcomes | 7 | | | 3 | | | 0 | | |
| N non-hierarchised outcomes | 8 | | | 0 | | | 18 | | |
| **Authors' conclusion at M3** | **Success** | | | **Non-conclusive** | | | **Success** | | |
|  | **Outcome nature** | **Variable name** | **Measure of association (CI95%)** | **Outcome nature** | **Variable name** | **Measure of association (CI95%)** | **Outcome nature** | **Variable name** | **p-value (α = 0.05)†** |
| **Primary outcomes** | Behav. | N condom-less anal sex acts*** | aIRR: 0.56 (0.32-0.99) | Clin. | Pregnancy since program enrolment*** | aIRR: 0.43 (0.11–1.70) |  |  |  |
|  |  |  |  | Behav. | N condom-protected sex acts*** | aIRR: 1.48 (1.30–1.68) |  |  |  |
|  |  |  |  | Behav. | Current use of birth control other than con-doms*** | aOR: 1.60 (1.08–2.37) |  |  |  |
|  |  |  |  | Behav. | Absti-nence from penile-vaginal sex*** | aIRR: 0.82 (0.55–1.23) |  |  |  |
| **Secondary outcomes** | Behav. | N anal sex part-ners*** | aIRR: 1.16 (0.74-1.81) | Intention | Inten-tions to use con-doms in the fu-ture*** | aOR: 1.09 (0.79–1.52) |  |  |  |
|  | Behav. | N condom-less anal sex part-ners*** | aIRR: 1.09 (0.64-1.87) | Intention | Inten-tions to use non-condom birth control in the fu-ture*** | aOR: 1.93 (1.31–2.84) |  |  |  |
|  | Behav. | Sex under the influence of substan-ces*** | aIRR: 0.91 (0.41-1.99) | Intention | Inten-tions to be absti-nent in the fu-ture*** | aOR: 0.95 (0.67–1.35) |  |  |  |
|  | Behav. | Uptake of PrEP*** | aOR: 1.65 (0.12-23.19) |  |  |  |  |  |  |
|  | Behav. | Uptake of PEP*** | aOR: 1.81 (0.11-30.40) |  |  |  |  |  |  |
|  | Health-care | Testing for HIV*** | aOR: 1.18 (0.59-2.35) |  |  |  |  |  |  |
|  | Health-care | Testing for STIs*** | aOR: 1.45 (0.69-3.02) |  |  |  |  |  |  |
| **Non-hierarchised outcomes** | Accept. | Accepta-bility and satisfac-tion with the interven-tion | NST |  |  |  | Behav. | CAS acts in prior 3 months*** | p = 0.68 |
|  | Accept. | User satisfac-tion with the app | NST |  |  |  | Behav. | CAS partners in prior 3 months*** | p = 0.54 |
|  | Dose + Reach | Feasibi-lity of interven-tion delivery | NST |  |  |  | Behav. | Currently on PrEP*** | p < 0.001 |
|  | Usability | Informa-tion quality provided by the app | NST |  |  |  | Behav. | Health protective communica-tion*** | p = 0.48 |
|  | Usability | Product quality of the app | NST |  |  |  | Behav. | Condom use errors*** | p < 0.05 |
|  | Usability | Service quality of the app | NST |  |  |  | Behav. | Condom use failures*** | p = 0.70 |
|  | Perceived effecti-veness | Net benefit from using the app | NST |  |  |  | Intention | Condom use inten-tions*** | p = 0.34 |
|  | Context | Health-care barriers | NST |  |  |  | Self-efficacy | Condom use self-efficacy*** | p = 0.30 |
|  |  |  |  |  |  |  | Know. | HIV knowledge | p < 0.001 |
|  |  |  |  |  |  |  | Attitudes | Attitudes towards HIV preven-tion*** | p = 0.59 |
|  |  |  |  |  |  |  | Self-efficacy | Individual HIV prevention self-efficacy*** | p < 0.001 |
|  |  |  |  |  |  |  | Self-efficacy | Safe sex with partner self-efficacy | p = 0.84 |
|  |  |  |  |  |  |  | Self-efficacy | Confidence to get an HIV test*** | p < 0.01 |
|  |  |  |  |  |  |  | Self-efficacy | Confidence to get PrEP*** | p < 0.001 |
|  |  |  |  |  |  |  | Psycho-social | Perceived vulnerability to HIV*** | p = 0.09 |
|  |  |  |  |  |  |  | Psycho-social | Perceived PrEP candi-dacy*** | p < 0.001 |
|  |  |  |  |  |  |  | Health-care | Lifetime HIV test*** | p < 0.001 |
|  |  |  |  |  |  |  | Health-care | Lifetime STIs test*** | p < 0.001 |

†: Pre-test post-test mean comparison. ***: Effectiveness outcomes used to investigate the impact of different outcome analysis strategies on intervention success in Table 2 of the main paper. Blue shading indicates outcomes which are used within equally weighted and differentially weighted outcome strategies in Table 2 of the main paper. **Outcome nature:** Accept.: Acceptability; Behav.: behavioural; Clin.: Clinical; Healthcare: Healthcare utilisation; Know.: Knowledge. **Abbreviations:** M: months; N: number of; HIV: human immunodeficiency virus; IMB: Information-Motivation-Behavioural; App: mobile application; RCT: randomised controlled trial; CI95%: 95% Confidence Interval; STIs: sexually transmitted infections; PrEP: pre-exposure prophylaxis; PEP: post-exposure prophylaxis; CAS: condomless anal sex; NST: not statistically tested.
