# Supplementary material for: A scoping review of outcome selection and accuracy of conclusions in complex digital health interventions for young people (2017–2023): methodological proposals for population health intervention research
Source: BMC Med. 2025 Jul 2;23:400. doi: 10.1186/s12916-025-04245-1 (PMC12224660; doi:10.1186/s12916-025-04245-1)
Supplement: Supplementary file 10 — Additional file 10: Fig. S2. Risk-of-bias assessment (ROB-2) for randomised controlled trials. Fig. S3. Risk-of-bias assessment (ROBINS-I) for non-randomised studies. [file 12916_2025_4245_MOESM10_ESM.docx]

## Additional File 10. Risk-of-bias assessment for articles presenting primary results on effectiveness

**Figure S2. Risk-of-bias assessment with Cochrane ROB-2 tool for interventions evaluated through randomised controlled trials (n=23)**

**Figure S3. Risk-of-bias assessment with Cochrane ROBINS-I tool for interventions evaluated through non-randomised studies (n=3)**
